# Serum metabolomic biomarkers of perceptual speed in cognitively normal and mildly impaired subjects with fasting state stratification

**DOI:** 10.1038/s41598-021-98640-2

**Published:** 2021-09-23

**Authors:** Kamil Borkowski, Ameer Y. Taha, Theresa L. Pedersen, Philip L. De Jager, David A. Bennett, Matthias Arnold, Rima Kaddurah-Daouk, John W. Newman

**Affiliations:** 1 West Coast Metabolomics Center, Genome Center, University of California Davis, Davis, CA 95616 USA; 2 Dept Food Science and Technology, University of California - Davis, Davis, CA 95616 USA; 3 Center for Translational and Computational Neuroimmunology, Department of Neurology and Taub Institute for Research on Alzheimer’s Disease and the Aging Brain, Columbia University Irving Medical Center, New York, NY 10032 USA; 4 Rush Alzheimer’s Disease Center, Rush Medical College, Rush University, Chicago, IL 60612 USA; 5 Department of Psychiatry and Behavioral Sciences, Duke Institute for Brain Sciences and Department of Medicine, Duke University, Durham, NC 27708 USA; 6 Institute of Computational Biology, Helmholtz Zentrum München - German Research Center for Environmental Health, Neuherberg, Germany; 7 Western Human Nutrition Research Center, United States Department of Agriculture - Agriculture Research Service, Davis, CA 95616 USA; 8 Department of Nutrition, University of California - Davis, Davis, CA 95616 USA

**Keywords:** Risk factors, Cognitive ageing, Dementia, Alzheimer's disease

## Abstract

Cognitive decline is associated with both normal aging and early pathologies leading to dementia. Here we used quantitative profiling of metabolites involved in the regulation of inflammation, vascular function, neuronal function and energy metabolism, including oxylipins, endocannabinoids, bile acids, and steroid hormones to identify metabolic biomarkers of mild cognitive impairment (MCI). Serum samples (n = 212) were obtained from subjects with or without MCI opportunistically collected with incomplete fasting state information. To maximize power and stratify the analysis of metabolite associations with MCI by the fasting state, we developed an algorithm to predict subject fasting state when unknown (n = 73). In non-fasted subjects, linoleic acid and palmitoleoyl ethanolamide levels were positively associated with perceptual speed. In fasted subjects, soluble epoxide hydrolase activity and tauro-alpha-muricholic acid levels were negatively associated with perceptual speed. Other cognitive domains showed associations with bile acid metabolism, but only in the non-fasted state. Importantly, this study shows unique associations between serum metabolites and cognitive function in the fasted and non-fasted states and provides a fasting state prediction algorithm based on measurable metabolites.

## Introduction

Neurocognitive disorders including Alzheimer’s dementia (AD) are associated with cognitive decline. Biochemical markers of altered cognitive capacity may provide diagnostic and prognostic biomarkers of these diseases and their associated metabolic trajectories before clinical symptoms manifest. Additionally, such biomarkers could provide new insights into the mechanisms of cognitive decline. Cognition can be decomposed into dissociable domains, characterized as perceptual speed, perceptual orientation along with semantic, working and episodic memory. These cognitive domains become increasingly inter-correlated as people become cognitively impaired^1^, and have been linked to pathologic changes in the brain^2^. While the events which initiate these changes are as yet unknown, dysregulated cellular mechanisms associated with metabolic dysfunctions and/or inflammatory responses are attractive hypotheses.

It has recently become clear that cardiometabolic disorders and associated low-grade systemic inflammation and altered lipid and energy metabolism, are risk factors for cognitive impairment^3–5^. Additionally, cardiometabolic disorders and cognition share circulating biomarkers and risk factors^6^. Our primary hypothesis was that cognitive decline is associated with an abrogation of normal epoxy fatty acid status based on findings that inhibition of soluble epoxide hydrolase, an enzyme previously implicated in the regulation of microvascular tone and inflammation, protects hippocampal neuronal death and reduces cognitive decline in mice^7^, and that genetic deletion of this enzyme slows amyloid beta associated Alzheimer’s disease onset in mice^8^. Further studies have shown that sEH inhibition reduces neuroinflammation^9^ and alpha-synuclein aggregation^10,11^, pathological feature of multiple neurocognitive disorders^12^.

Therefore, changes in circulating markers of low-grade inflammation and metabolism may track these pertinent metabolic changes. Obesity and the metabolic syndrome shift the profile of both plasma lipids and multiple lipid-derived physiological mediators^13,14^. Four important families of such lipid mediators readily detected in the circulation are the oxygenated polyunsaturated fatty acids (i.e. oxylipins), the endogenous cannabinoid receptor activators and their structural equivalents (i.e. endocannabinoids), bile acids and steroids.

The oxylipins including fatty acid alcohols, diols, epoxides, ketones, and prostanoids are derived from multiple polyunsaturated fatty acids (PUFA) by the action of cyclooxygenases (COX), lipoxygenases (LOX), cytochrome P450 (CYP), soluble epoxide hydrolase (sEH) or reactive oxygen species (ROS) and various downstream enzymatic processes^15,16^. Circulating endocannabinoids are produced either by acylation and release of acyl ethanolamides from phosphatidylethanolamine, or as a product of glycerol-lipid metabolism (monoacylglycerols).

Oxylipins and endocannabinoids are known to regulate multiple processes including both acute and low-grade systemic inflammation^16,17^, cardiovascular health^18^, neuronal outgrowth, cell differentiation and energetics^19^. Bile acids and steroid are also linked to the regulation of glucose and insulin metabolism^20^, energy metabolism and inflammation^21,22^ and implicated in the pathogenesis of type 2 diabetes and metabolic syndrome^23^. Previous studies reported associations between, cognition and plasma levels of oxylipins^24^, bile acids^25,26^ and steroids^27,28^. However, broader simultaneous assessments of lipid mediator profiles in the context of mild cognitive impairment have not been conducted to date.

Frozen collections of serum and plasma from studies of neurocognitive disorders, including measures of cognitive function, provide a resource for biomarker discovery in this area^29^. However, opportunistically collected samples rarely contain information regarding fed/fasted states, which can compromise “omics” analyses. Here, we took advantage of data and biospecimens from subjects in the Religious Order Study and Rush Memory and Aging Project (ROS/MAP)^30^, develop a predictive tool for the fasted/non-fasted state discrimination and stratify our biomarker discovery effort by the fasted state. We describe an exploration of circulating oxylipin, endocannabinoids, bile acids, and steroids for biomarkers of cognitive impairment, providing insights into unique associations in basal and postprandial metabolism.

## Materials and methods

### Subjects

Participants in the Religious Orders Study (ROS) are older nuns, priests, and brothers from across the United States, while those in the Rush Memory and Aging Project (MAP) are older lay persons from the greater Chicago area^30^. Both studies enrolled persons without known dementia and perform annual detailed clinical evaluations. Both studies were approved by an Institutional Review Board of Rush University Medical Center. All methods were performed in accordance with the relevant guidelines and regulations required of an Institutional Review Board of Rush University Medical Center which approved the project. All participants signed an informed consent and a repository consent to allow their biospecimens and data to be shared. ROS/MAP resources can be requested at www.radc.rush.edu. The current sample consists of 198 subjects with 14 subjects having two blood samples collected on average 5.8 ± 3.3 years apart. Repeated blood draws were in opposite fasting states (either fasted or non-fasted). Subjects demographics: 22% male, 95% white and non-Hispanic. Average age (mean ± standard deviation) = 78.2 ± 7.2, average BMI = 27.2 ± 4.8 average years of education = 15.3 ± 2.8. Number of known fasted samples as recorded by a technician = 59; non fasted = 80, unknown = 73.

### Clinical evaluation of cognition

All subjects are under a yearly structured clinical evaluation, including a medical history, neurologic examination and cognitive testing. A battery of 21 tests was performed in each study with 19 in common. The MMSE was used for descriptive purposes, except for ten items which informed on orientation^31^. One test, Complex Ideational Material, was also only used in diagnostic classification^32^. The remaining 17 tests were used to create a global measure included seven measures of episodic memory: the ten item Word List Memory, Word List Recall, Word List Recognition from CERAD^33^, and a 12-item- immediate and delayed recall of the East Boston story^34^, Story A from Logical Memory^35^. Semantic memory was assessed with a 15-item form of the Boston Naming Test from CERDA^36^, animals, and fruits and vegetable verbal fluency^37^, and a word reading test^38–40^. Working memory was assessed with Digit Ordering and Digit Span Forward and Backward^35^, and Digit Ordering^41–43^. Perceptual speed was assessed with the Symbol Digit Modalities Test^44^, and Number Comparisons^45^. Visuospatial ability was assessed with a 15-item version of Judgment of Line Orientation^46^, and an 11-item version of Standard Progressive Matrices^47^. Eleven of the tests were used to inform on clinical judgement of cognitive impairment, dementia and Alzheimer’s dementia in a multi-step process^48,49^. Some of the tests were modified from their original format and further details can be found and obtained at https://www.radc.rush.edu. Seventeen tests are used for measure of global cognition and five distinct cognitive domains including perceptual speed, perceptual orientation, episodic memory, semantic memory and working memory^50^. The global cognition was calculated by converting each test to a z score based on the mean and standard deviation and averaging the 17 tests; the domains were created by averaging subsets of z-scores as previously reported in detail^50^. The subject’s biometrics and cognition assessments together with composite cognition scores and indication of cognitive tests used to generate each cognitive domain, stratified by gender and fasting state, are provided in the Supplemental Table S1.

### Quantification of clinical lipids, glucose and glycosylated hemoglobin

Phlebotomists and nurses collected the blood specimen as previously reported^51^. Tests were performed by Quest Diagnostics (Secaucus, NJ). For this study we used glucose (mg/dL), hemoglobin A1c, expressed as a percentage of hemoglobin, and a basic lipid panel consisting of total cholesterol, HDL and LDL cholesterol, and triglycerides (all units mg/dL).

### Quantification of oxylipins, endocannabinoids, PUFA, non-steroidal anti-inflammatory drugs, bile acids and steroids

Serum concentrations of non-esterified PUFA, oxylipins, endocannabinoids, a group of non-steroidal anti-inflammatory drugs (NSAIDs) including ibuprofen, naproxen, acetaminophen, a suite of conjugated and unconjugated bile acids, and a series of glucocorticoids, progestins and testosterone were quantified by liquid chromatography tandem mass spectrometry (LC–MS/MS) after protein precipitation in the presence of deuterated metabolite analogs (i.e. analytical surrogates) using published procedures^52,53^. Samples were processed with rigorous quality control measures including the analysis of batch blanks and replicates of serum pools and NIST Standard Reference Material 1950 (Sigma-Aldrich). Samples were re-randomized for acquisition, with method blanks and internal reference material and calibration sets scattered regularly throughout the set. Instrument limits of detection (LODs) and limits of quantification (LOQs) were estimated according to the Environmental Protection Agency method (40 CFR, Appendix B to Part 136 revision 1.11, U.S. and EPA 821-R-16-006 Revision 2). Briefly, calibration standards were analyzed in triplicate and differences in measured mean concentrations between levels were tested using a t-test at α = 0.05. The first concentration with a significant difference from its next lowest level was established as the first detectable standard, and the LOD was estimated as the standard deviation in that average concentration multiplied by 2.35, the 1-tailed t-distribution at a 95% with 2-degrees of freedom. The LOQ was established at 3-times the LOD. These values were then transformed into sample nM concentrations by multiplying the calculated concentration by the final sample volume (i.e. 250 µL) and dividing by the volume of sample extracted (i.e. 50 µL). A complete analyte list with their LOD and LOQ is provided in the Supplemental Table S2. The majority of analytes were quantified against analytical standards with the exception of eicosapentaenoyl ethanolamide (EPEA), palmitoleoyl ethanolamide (POEA), and the measured PUFA [i.e. linoleic acid (LA); alpha-linolenic acid (aLA); arachidonic acid (AA); eicosapentaenoic acid (EPA); docosahexaenoic acid (DHA)]. For those compounds the area counts were recorded, adjusted for deuterated-surrogate and the relative response factors were expressed as the relative abundance across all analyzed samples. MAGs are reported as the sum of 1- and 2- isomers, due to their potential isomerization during the sample processing. The complete metabolomic data are available via the AD Knowledge Portal (https://adknowledgeportal.synapse.org). The AD Knowledge Portal is a platform for accessing data, analyses, and tools generated by the Accelerating Medicines Partnership (AMP-AD) Target Discovery Program and other National Institute on Aging (NIA)-supported programs to enable open-science practices and accelerate translational learning. The data, analyses and tools are shared early in the research cycle without a publication embargo on secondary use. Data is available for general research use according to the following requirements for data access and data attribution (https://adknowledgeportal.synapse.org/DataAccess/Instructions). See 10.7303/syn22344904. Targeted metabolomics was used as authors seek to quantify specifically lipid mediators, with the serum concentration of some metabolites at the pM levels. Targeted technique provides much lower limits of detection due to the signal purification and the known targets allow hypothesis—based experimental design. Additionally, absolute quantification allows cross—cohort comparison and utilization of the fasting state predictive model in the other studies.

### Statistical analysis

All statistical tests were performed using JMP Pro 14 (JMP, SAS institute, Carry, NC). Prior to analysis, two data points were removed as outliers using the robust Huber M test and missing data were imputed using multivariate normal imputation for variables which were at least 75% complete. Imputation facilitated multivariate data analysis and did not significantly influence univariate results. Additionally, variables were normalized, centered and scaled using Johnson’s transformation, with normality verification using the Shapiro–Wilk test. Cognitive sores were adjusted for BMI, sex, age, race and education and their residuals were used for further analysis. Metabolite inter-correlations were evaluated using Spearman’s rank-order correlations. Variable clustering by hierarchical cluster analysis used the Ward agglomeration. Multiple comparison control was accomplished with the false discovery rate (FDR) correction method of Benjamini and Hochberg^54^, with the number of independent observations determined by the correlative structure of variables (number of variable clusters).

Predictive models for fasting state and cognitive functions were prepared using a combination of bootstrap forest and stepwise linear regression modeling, with Bayesian information criterion (BIC) cutoff. Variable selection by bootstrap forest was used to minimize the effect of outliers. Variables most frequently appearing in the models were identified by bootstrap forest (logistic or regression, respectively): trees in forest = 100; terms sampled per split = 5; bootstrap sample rate = 1. A variable contribution scree plot was generated using variable rank and the likelihood ratio of chi-square (for categorical fasted/non-fasted prediction) or sum of squares (for continues cognitive scores). The scree plot was used to determine a likelihood ratio of chi-square or sum of squares cutoff value for variables contributing to the model. Selected variables were then subjected to forward stepwise logistic regressions for fasted/non-fasted predictions, or forward stepwise linear regressions for cognitive scores. Data were split into training (60%) and validation (40%) cohorts, with balanced separation across metabolites and cognitive domains. Stepwise analysis was performed with the maximal validation r^2^ as the model stopping criteria, or if an additional step increased the BIC. Stepwise regression was used to highlight independent predictors of cognitive domains.

## Results

### Serum lipid mediators predict the fasting state

**Figure 1:**
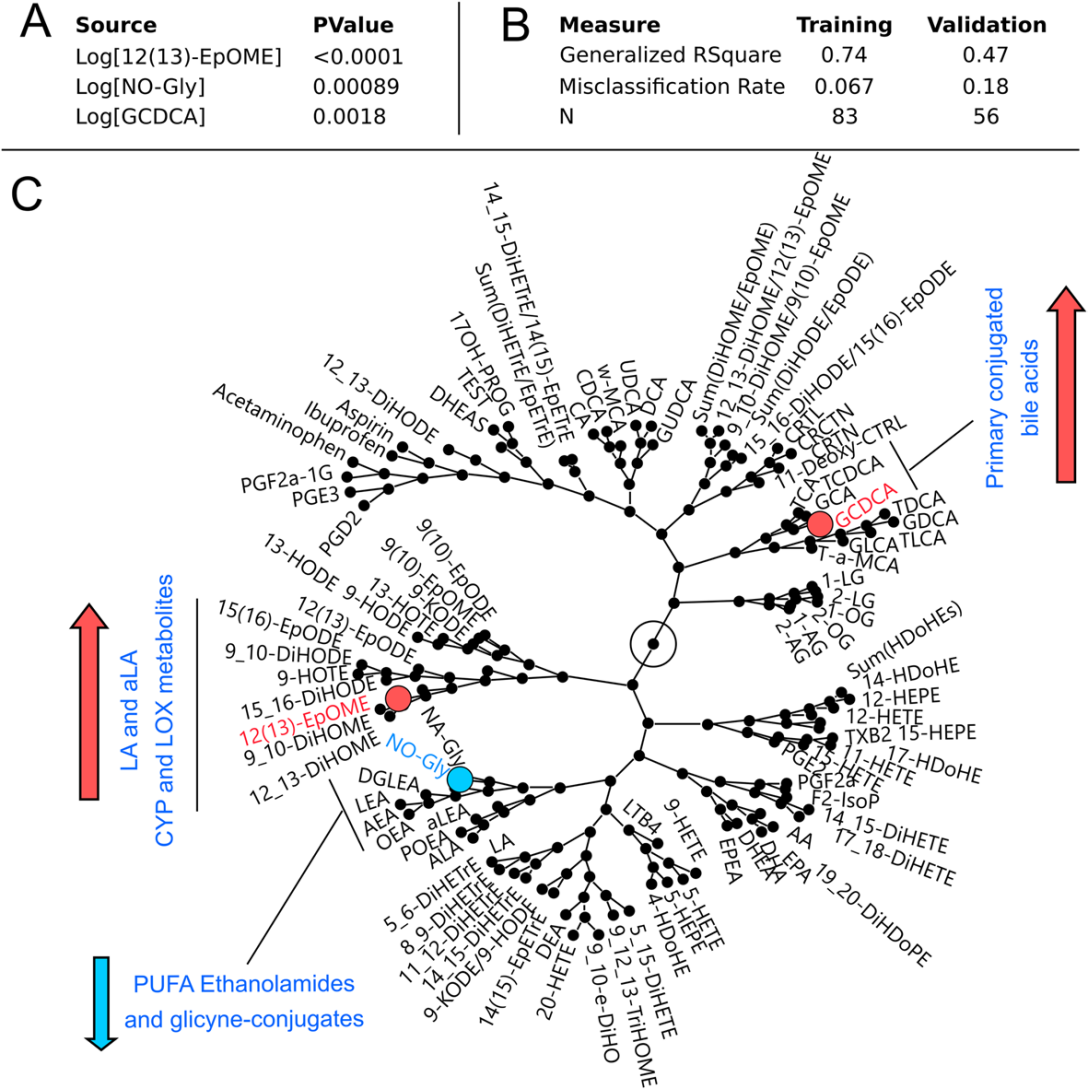
Serum lipid metabolites and bile acids are predictors of the fasting state. (**A**) Stepwise logistic model parameters predicting the fasting state using 12(13)-EpOME, GCDCA and NO-Gly. (**B**) Model statistics. (**C**) Visualization of the correlative environment (generated using hierarchical clustering) of metabolites used for fasting state prediction. Nodes represent branching points in the hierarchical clustering network with metabolites on the fringe named. Metabolite used in the final model are indicated by colors. Directionality of changes in metabolites due to non-fasted state compared to the fasted state are indicated by arrows.

Our cohort consists of 212 samples including 59 fasted, 80 non-fasted and 73 of unknown fasting state. Using samples with known fasting state, a fasting state prediction model was developed using measured PUFA, lipid mediator, bile acid, steroid, clinical lipid and glucose data. Prior to analysis, subjects were randomly assigned to the training (60%, n = 83) and validation (40%, n = 56) cohorts. Clinical lipids (e.g. triglycerides or cholesterol) and glucose did not produce strong predictive models and did not contribute to the final model. A high probability of the fasted state was described by low levels of the LA-derived CYP metabolite [12(13)-EpOME], low levels of the primary conjugated bile acid glycochenodeoxycholic acid (GCDCA) and elevated levels of the glycine-conjugated oleic acid (NO-Gly; Fig. 1A,B). The model misclassification rate was 12%., with fasting probability described by the Eqs. (1) and (2).

1 $$Probability\;for\;fasted= \frac{1}{(1+Exp\;(-Lin.\;prob.\;fasted))}$$

Equation 1. Probability of the fated state. Where “Lin.prob.fasted” is defined by the Eq. (2):

2 $$Lin.\;prob.\;fasted=10.01-\left(2.82\times a\right)+\left(1.94\times b\right)-(1.35\times c)$$

Equation (2). Lin prob fasted: *a* = Log[12(13)-EpOME]; *b* = Log(NO-Gly); *c* = Log(GCDCA). Concentrations expressed in (nM).

Oxylipins, endocannabinoids, PUFA, bile acids and steroids create correlative structures along metabolic pathways or from common precursor fatty acids (Fig. 1C). Therefore, similar fasting state predictions could be achieved by substituting metabolites with ones close in the correlation network. For example, NO-Gly can be effectively replaced by oleoyl ethanolamide (OEA). Validation of model was performed using an independent cohort^55^ of fasted plasma (n = 133) and showed a misclassification rate of 17%, dropping to 12% when considering samples with a probability of prediction > 70%. To facilitate understanding of oxylipin and endocannabinoid metabolic relationship, their synthesis pathway from PUFA as well as coverage of metabolites detected in this study are presented in the Supplemental Fig. S1.

### Fasted and non-fasted serum reveal distinct associations between lipid mediators and cognitive functions

**Figure 2:**
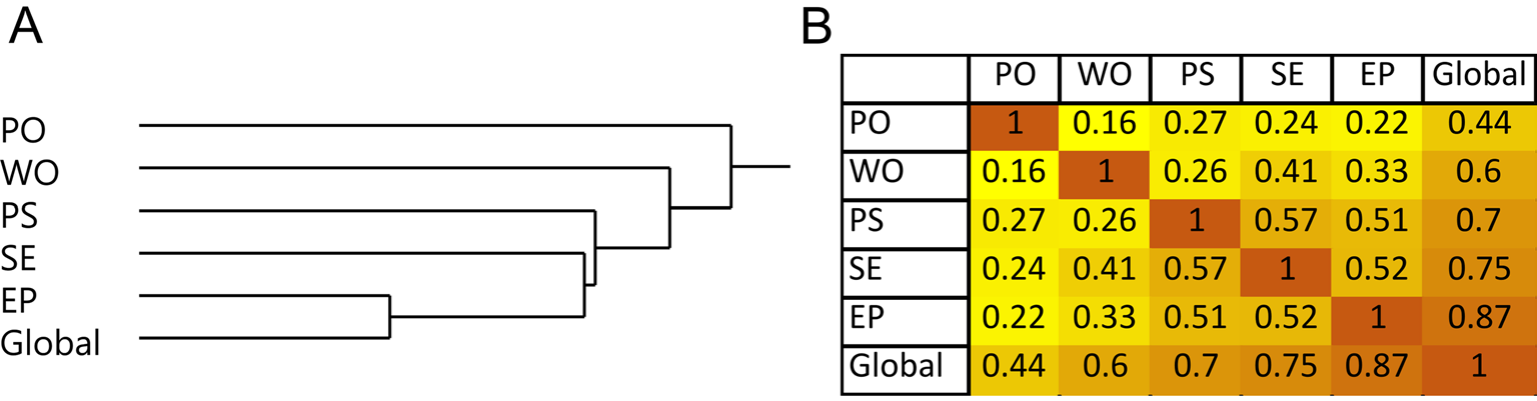
Correlative relationships between cognitive domains. (**A**) Hierarchical clustering of cognitive domains using Ward method. (**B**) Pearson’s correlation matrix. *PO* perceptual orientation, *WO* working memory, *PS* perceptual speed, *SE* semantic memory, *EP* episodic memory, *Global* global cognition.

Spearman’s rank correlations demonstrated associations between serum lipid mediators and cognitive functions. Cognitive scores were adjusted for BMI, gender, age, race and education. The analysis was stratified by subject fasting states. Figure 2 shows correlation between the five cognitive domains.

Oxylipins and endocannabinoids showed the greatest number of associations with perceptual speed (from 8 to 10% of metabolites in fasted and non-fasted samples respectively, Table 1). The number of associations for other cognitive domains and global cognition did not exceed 5% of the measured oxylipins and endocannabinoids (Supplemental Table S3).

**Table 1:** Spearman’s rank order correlations between serum oxylipins and endocannabinoids and perceptual speed.

Metabolite	Non-Fasted (n = 141)	Fasted (n = 71)
**Fatty acids, ethanolamides and hydroxy fatty acids**		
LA	0.25	
AA		0.26
EPA	0.22	
DHA	0.25	
EPEA	0.18	
POEA	0.24	
4-HDoHE	0.18	
15-HEPE	0.2	
**Dihydroxy fatty acids—sEH pathway**		
14,15-DiHETE		− 0.27
19,20-DiHDoPE	0.2	− 0.31
Sum (n3-Diols)		− 0.28
Sum (DiHETEs)		− 0.25
12,13-DiHOME/ 12(13)-EpOME		− 0.32
**Prostaglandins—COX pathway**		
PGD2		0.25

The numbers represent Spearman’s ρ with the p value < 0.05 and FDR corrected with the q = 0.2. Full names of all identified compounds are presented in the Supplemental Table S2 and correlation for all cognitive domains are presented in the Supplemental Table S3.

Fasted and non-fasted samples showed distinct correlation patterns. In non-fasted subjects perceptual speed was positively associated with the level of free PUFA, particularly LA, eicosapentaenoic acid (EPA) and docosahexaenoic acid (DHA), as well as the N-acyl ethanolamides derived from palmitoleate (POEA), and EPA (EPEA) and the EPA- and DHA-derived mono-alcohols (15-HEPE and 4-HDoHE respectively). These associations were absent in fasted subjects. Additionally, when fasted and non-fasted subjects were analyzed together without fasting state stratification, the above-mentioned associations were either not present or weaker than in non-fasted subjects alone, see Supplemental Table S4).

On the other hand, fasted samples manifested negative correlations between perceptual speed and sEH products of EPA and DHA, and the ratio of LA vicinal diols (i.e. those with two hydroxy groups on adjacent carbons) to their corresponding epoxides, an estimator of sEH activity^56^. This association was not detected in non-fasted subjects. Importantly, the cognitive domains scores were not different between the fasting states. Additionally, interaction with sex were not detected for the above-mentioned associations.

Numerous significant correlations were detected between bile acid levels and cognitive scores, mainly in non-fasted subjects (episodic memory: 9% to 38%; semantic memory: 3% to 25%; global cognition: 6% to 25%; and perceptual speed: 3% to 16% in fasted and non-fasted subjects respectively, Table 2). Perceptual orientation and working memory showed < 6% associations (Supplemental Table S3).

**Table 2:** Spearman’s rank order correlations between serum bile acids and cognitive domains.

Metabolite	Non-Fasted (n = 141)				Fasted (n = 71)			
Cognitive domain	PS	SE	EP	Global	PS	SE	EP	Global
**Bile Acids—unconjugated**								
CDCA	0.2	0.19					0.27	
DCA		0.2						
**Bile Acids—conjugated**								
TCDCA		− 0.2						
TLCA	− 0.2	− 0.27	− 0.28	− 0.29				
TDCA			− 0.18					
GDCA			− 0.21					
**Bile acids—conjugated/unconjugated**								
TDCA/DCA		− 0.25	− 0.18	− 0.22				
GDCA/DCA		− 0.3	− 0.23	− 0.27				
GCDCA/CDCA	− 0.24	− 0.28		− 0.2				
GCA/CA			− 0.22					
TCA/CA			− 0.21					
GUDCA/UDCA							0.3	0.32
**Bile acids—glycine/taurine**								
(GDCA + GLCA)/ (TDCA + TLCA)			0.18	0.19				
**Bile Acids—dehydroxylation by bacteria**								
TDCA/CA			− 0.26	− 0.19				
GDCA/CA			− 0.28	− 0.18				
DCA/CA			− 0.19					
GLCA/CDCA	− 0.19							
TLCA/CDCA	− 0.24	− 0.28	− 0.24	− 0.27				
**Bile Acids—other**								
T-a-MCA					− 0.28	− 0.26	− 0.29	− 0.31
T-a-MCA/CDCA	− 0.22	− 0.27		− 0.2		− 0.26	− 0.35	− 0.31
w-MCA/T-a-MCA		0.23		0.2		0.28		

*PS* perceptual speed, *SE* semantic memory, *EP* episodic memory, *Global* global cognition.

The numbers represent Spearman’s ρ with the p value < 0.05 and FDR corrected with the q = 0.2. Full names of all identified compounds are presented in the Supplemental Table S2 and correlation for all cognitive domains are presented in the Supplemental Table S3.

In non-fasted subjects, unconjugated bile acids correlated positively with perceptual speed and semantic memory. On the on the other hand, conjugated bile acids and the ratios of conjugated to unconjugated bile acids showed negative associations with perceptual speed, semantic and episodic memory and global cognition. Additionally, positive associations were observed between the ratio of glycine to taurine conjugated bile acids and episodic memory and global cognition. Negative associations were observed between the ratio of the downstream product to their precursor—cholic acid (CA) and episodic memory and global cognition. Finally, negative associations were observed between the ratio of tauro-alpha-muricholic acid (T-a-MCA) and its precursor chenodeoxycholic acid (CDCA).

Few associations between cognition and bile acids were observed in the fasted subjects. Negative associations were observed between T-a-MCA and T-a-MCA/CDCA ratio and episodic and semantic memory, perceptual speed and the global cognition. Also, positive associations were observed between the ratio of glycine conjugated to unconjugated ursodeoxycholic acid (UDCA) and episodic memory and global cognition. No associations were found between cognitive domains and steroid hormones.

### Fasted state lipid mediators predict perceptual speed

**Figure 3:**
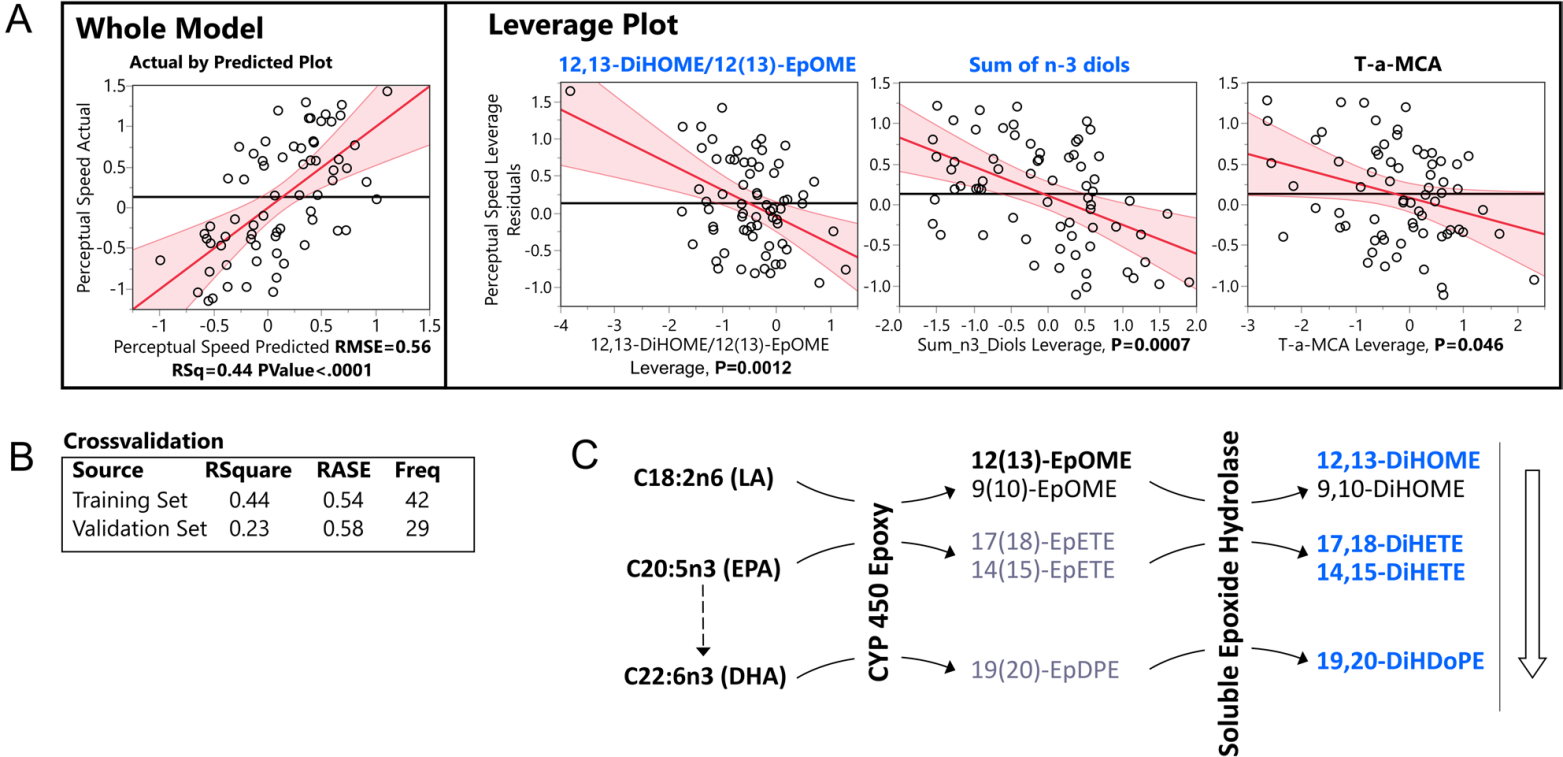
Least square regression model of perceptual speed. (**A**) Actual by predicted plot of a whole model and leverage plots of model components. (**B**) Model cross-validation statistics using training set (60%, n = 44) and validation set (40%, n = 33). (**C**) Model components of soluble epoxide hydrolase metabolism projected onto their metabolic pathway. Metabolic pathway starts with the fatty acids on the left, farther, metabolizing enzymes are indicated on the arrows. Multiple possible metabolites of the pathway are indicated. Metabolites of sEH used for the model are highlighted. Color of the metabolites as well as an arrow next to the metabolic pathway represents directionality of the correlation with perceptual speed (orange—positive, blue—negative). *RMSE* root mean squared error, *LA* linoleic acid, *CYP 450* cytochrome p450, *sEH* soluble epoxide hydrolase, *EpOME* epoxy octadecanoic acid, *DiHOME* dihydroxy octadecanoic acid, *EpETE* epoxy eicosatrienoic acid, *DiHETE* dihydroxy eicosatrienoic acid, *EpDPE* epoxy docosapentaenoic acid, *DiHDoPE* dihydroxy docosapentaenoic acid.

Predictive models revealed covariate relationships between serum lipid mediators and cognition. Stepwise linear regression models (Supplemental Table S5) were built independently for each cognitive domain and for fasted/non-fasted samples. Valid models could not be generated using non-fasted subject data. Consistent with Spearman’s correlation results, perceptual speed formed the strongest model (R^2^_perceptual speed_ = 0.44; R^2^_perceptual orientation_ = 0.4; R^2^_episodic memory_ = 0.29; R^2^_global cognition_ = 0.24) using samples from fasted subjects. The final model for perceptual speed is presented in the Fig. 3. This model included the ratio of LA-derived 12,13-DiHOME to 12(13)-EpOME, the sum of n-3 diols, consisting of EPA- and DHA-derived diols (14,15-DiHETE, 17,18-DiHETE and 19,20-DiHDoPE) and T-a-MCA. The epoxide/diol ratio and the sum of n-3 diols contributed the most to the model, with p-values of 0.0012 and 0.0007 respectively, and T-a-MCA with a weaker, but significant contribution (p value = 0.046). Supplemental Fig. S2 shows correlative structure of all detected metabolites in fasted subjects. Sum of n-3 diols consist of all detected EPA and DHA diols. Corresponding EPA and DHA-derived epoxides were not detected. Additionally, to further illustrate the correlative structure of the data, variables preselected for the stepwise model were subjected to variable clustering and three clusters were generated out of 12 variables subjected to the stepwise model (Supplemental Table S6). Each variable that forms the model belongs to separate cluster. Valid and similar predictive model for PS can be achieved by utilizing the cluster components (average of all variables in the cluster, Supplemental Fig. 4), further pointing towards three independent groups of predictors for the PS.

## Discussion

In the current study we identified serum lipid mediators associated with cognitive function in a cohort exhibiting normal to mildly-impaired cognition. MCI has multiple sources including normal cognitive decline, vascular dementia^57^ and a variety neuropathologies, including AD^58^. While the vast majority of Alzheimer’s dementia cases have AD at autopsy^48^, only about two thirds of MCI have AD^49^. Further, in both cases AD often co-exists with other brain pathologies^59^. Moreover, this study provides a solution to the unknown fasting state of subjects that may occur when using opportunistically collected samples and identifies unique associations with cognition in both fasted and non-fasted states.

Opportunistically collected serum and plasma are often collected without regards an individuals’ fasting state, compromising investigations probing peripheral factors influenced by postprandial fluctuations in the metabolome^60^, proteome^61^ and transcriptome^62^. Using metabolomic data, we have developed a tool to determine subject fasting states and show enhanced statistical power with fasting state stratification. In addition, fasting state stratification highlighted aspects of metabolism which manifest themselves uniquely in the postprandial and fasted states. Indeed, while fasted serum has been a source of many markers for metabolic diseases^63^, individual responses to a meal can carry information regarding metabolic flexibility^64^, prediabetes state^65^ or postprandial inflammation^66^. To our knowledge, the issue of the mixed population of fasted and non-fasted subjects in the biobanked samples has not been previously addressed. As our model was built using absolute quantification it is transferable to other studies and could be especially useful for cohorts without fasting state information. Of note, the stability of metabolomics factors used to generate the fasting state predictive model during sub-optimal collection practices (i.e. storage at room temperature for days prior to refrigeration)^67^ and upon prolong freezer storage were previously described^68^.

The postprandial state is the dominant metabolic state due to the common ingestion of multiple meals yielding 6–8 h postprandial fluctuation in lipoprotein particles^69^, non-esterified lipids^70^, hormones^60^, etc. The strongest positive associations in the non-fasted samples were observed between perceptual speed and levels of non-esterified LA, EPA, DHA, the 15-LOX metabolite of EPA (15-HEPE) and palmitoleate- and EPA-derived ethanolamides (i.e. POEA and EPEA). Other measured ethanolamides did not show significant associations with perceptual speed. The positive association between LA and perceptual speed suggests a role of LA in regulating memory domains, consistent with studies showing reduced LA concentrations in multiple brain regions affected by Alzheimer’s Disease^71^. Although decrease in the fasting polyunsaturated fatty acids was previously reported to be associated with cognition^72^ and AD^73^, to our knowledge no study linked postprandial fatty acids metabolism and cognition.

Ethanolamides are generally considered anti-inflammatory^74^ and neuroprotective^75^, however, their postprandial physiological consequences are not well understood. Like PUFA, all ethanolamides are lower in non-fasted versus fasted subjects (Supplemental Fig. S3), consistent with the literature^76^, further validating developed predictive model. This may suggest that maintaining a higher level of LA and/or POEA and/or EPEA in the postprandial state may reflect metabolism beneficial to perceptual speed and cognition and is not dependent on the “basal” fasted state. The majority of ethanolamide studies have focused on derivatives of AA, oleic acid and palmitic acid, i.e. AEA, OEA and PEA respectively. AEA and PEA can activate CB1 and CB2 receptors^77^, important players in neuroinflammatory processes^78^. Moreover, AEA can similarly activate the transient vanilloid receptor type 1 (TRPV1) involved in the transduction of acute and inflammatory pain signals in the periphery^79^, and have a variety of functions within the central nervous system, and may mediate some excitotoxic effects^80^. OEA, a peroxisome proliferator-activated receptor α agonist, is a regulator of satiety and sleep with both central and peripheral anorexigenic effects^77^. Similarly, a satiety effect was achieved by external administration of the linoleoyl ethanolamide (LEA) and α-linolenoyl ethanolamide (aLEA) respectively^81^. However, little is known about the biological actions of POEA and EPEA. Additionally, palmitoleic acid and its metabolites are highly abundant in adipose tissue and have been described adipose derived lipokines^82^, which may indicate a specific involvement of adipose tissue in the maintenance of perceptual speed.

In the non-fasted state, bile acids manifested similar relationships with perceptual speed, semantic and episodic memory and global cognition. Generally, cognitive domains showed positive associations with unconjugated and negative associations with both taurine and glycine conjugated bile acids, the observation strengthened by associations with conjugated/unconjugated bile acid ratios, implying a role for liver metabolism in cognitive maintenance. Of note, the same associations were observed for primary and secondary bile acid. Additionally, we saw negative associations of episodic memory and global cognition with the ratio of both conjugated and unconjugated deoxycholic acid (DCA) to cholic acid (CA) and conjugated lithocholic acid (LCA) to CDCA. Those ratios represent dihydroxylation of primary bile acids (CA and CDCA) by gut bacteria and were previously reported to be negatively associated with cognition^83^ and atrophy, and brain glucose metabolism in AD^84^.

These findings suggest increased liver bile acid modification (i.e. conjugation with amino acids), as well as gut microbiome activity may negatively influence cognition. Importantly, these relationships were not observed in fasted samples, suggesting the importance of postprandial metabolism to either drive or highlight these metabolic associations with cognition, warranting further clinical trials using standardized meal tolerance tests. Standard meal tolerance test is routinely used to assess metabolic flexibility and postprandial inflammation, a critical factor for cardiovascular health. It is based on the idea that metabolic features can be reviled during metabolic stress, i.e. meal challenge^85^. Additionally, postprandial inflammation is now shown to be an important factor for atherosclerosis and vascular dysfunction^86,87^, factors contributing to cognitive impairment^88^.

Using only fasted subjects, we found perceptual speed to be negatively associated with sEH activity reflected by LA-dependent product: substrate ratios^56^, EPA- and DHA-derived sEH metabolites, and T-a-MCA and positively associated with the glycine conjugation ratio of UDCA (GUDCA/UDCA). Notably, and the predictive model for perceptual speed depended on both sEH activity assessments and sEH-derived omega 3 diols, these metabolic domains appear to contain independent information. Of note, addition of T-a-MCA provided only slight improvement to the model and in alternate iterations of the model through bootstrapping could be replace by free AA (positively associated with perceptual speed). Therefore, our results implicate eighteen carbon fatty acid metabolism (i.e. sEH action on LA and aLA epoxides) and long chain omega 3 fatty acid metabolism (i.e. sEH activity on EPA and DHA epoxides) in the decline of perceptual speed. This is an agreement with two recent studies which showed negative associations between circulating sEH activity and executive function^89,90^.

Epoxy fatty acids have potent vasorelaxant and anti-inflammatory properties, while fatty acid diols have demonstrated pro-inflammatory effects and actions as inhibiters of protein kinase B- (i.e. Akt) dependent processes^91^. Recent studies of mice and men have implicated sEH in neurodegenerative diseases of the brain^92^. Moreover, DHA feeding enhances the therapeutic efficacy of sEH inhibitors in reducing neurocognitive complications in rodent models of diabetes^93^. Together, these studies provide strong evidence that the identified shifts in sEH metabolism in association with cognitive decline may be linked to the underlying pathology of this process.

In contrast to the non-fasted state, in the fasted state general association between bile acids metabolism and cognition were not observed, and few specific bile acids showed significant correlations. The ratio of conjugated to unconjugated UDCA was positively associated with episodic memory and global cognition, whereas T-a-MCA was negatively associated with almost all cognitive domains. UDCA and its conjugated derivatives are hydrophilic bile acids previously reported to improve mitochondrial function^94^ and manifest neuroprotective properties both in vivo^95^ and prevent amyloid-β—induced neuronal death in vitro^96^. T-a-MCA appears in the predictive model for perceptual speed, together with sEH, suggesting their independent association with cognition. GUDCA/UDCA and T-a-MCA both appear in predictive model for episodic memory and global cognition, suggesting their independent associations with cognition.

In conclusion, here we have analyzed serum from the ROS/MAP cohort using a suite of targeted metabolomic assays in search of biomarkers of cognitive function with plausible links to inflammatory responses and energy metabolism. Our study suggests the involvement of sEH and omega-3 PUFA metabolism in cognition. Moreover, during the course of this effort we have produced a tool to determine subject fasting state when unknown and demonstrated the pivotal nature of this discrimination in biomarker discovery. We have demonstrated that the fasted and non-fasted states carry distinct information regarding the connection of metabolism and cognition. As opportunistically collected non-fasted samples manifest high variance and lack of control over the type and the time of the meal, future studies using a standardized mix meal tolerance test^97^ could prove useful to validate and discover new relationships between postprandial metabolism and cognition.

## Supplementary Information

Supplementary Information 1.

Supplementary Information 2.

Supplementary Information 3.

Supplementary Information 4.

Supplementary Information 5.

Supplementary Information 6.

Supplementary Information 7.

Supplementary Information 8.

Supplementary Information 9.

Supplementary Information 10.

## References

[1] Wilson RS, Segawa E, Hizel LP, Boyle PA, Bennett DA (2012). Terminal dedifferentiation of cognitive abilities. Neurology.

[2] Magistro D (2015). The relationship between processing speed and regional white matter volume in healthy young people. PLoS ONE.

[3] Alkan E (2019). Metabolic syndrome alters relationships between cardiometabolic variables, cognition and white matter hyperintensity load. Sci. Rep..

[4] Bosia M (2018). Improving cognition to increase treatment efficacy in schizophrenia: Effects of metabolic syndrome on cognitive remediation's outcome. Front. Psychiatry.

[5] Monthe-Dreze C, Rifas-Shiman SL, Gold DR, Oken E, Sen S (2019). Maternal obesity and offspring cognition: The role of inflammation. Pediatr. Res..

[6] Lai MMY (2020). Relationship of established cardiovascular risk factors and peripheral biomarkers on cognitive function in adults at risk of cognitive deterioration. J. Alzheimers Dis..

[7] Wu J, Fan Z, Zhao Y, Chen Q, Xiao Q (2021). Inhibition of soluble epoxide hydrolase (sEH) protects hippocampal neurons and reduces cognitive decline in type 2 diabetic mice. Eur. J. Neurosci..

[8] Lee HT, Lee KI, Chen CH, Lee TS (2019). Genetic deletion of soluble epoxide hydrolase delays the progression of Alzheimer's disease. J. Neuroinflammation..

[9] Ghosh A (2020). An epoxide hydrolase inhibitor reduces neuroinflammation in a mouse model of Alzheimer's disease. Sci. Transl. Med..

[10] Huang HJ, Wang YT, Lin HC, Lee YH, Lin AM (2018). Soluble epoxide hydrolase inhibition attenuates MPTP-induced neurotoxicity in the nigrostriatal dopaminergic system: Involvement of alpha-Synuclein aggregation and ER stress. Mol. Neurobiol..

[11] Ren Q (2018). Soluble epoxide hydrolase plays a key role in the pathogenesis of Parkinson's disease. Proc. Natl. Acad. Sci. USA.

[12] Saleh H (2015). Mini review: Linkage between alpha-Synuclein protein and cognition. Transl. Neurodegener..

[13] Shearer GC (2018). Abnormal lipoprotein oxylipins in metabolic syndrome and partial correction by omega-3 fatty acids. Prostaglandins Leukot Essent Fatty Acids.

[14] Grapov D, Adams SH, Pedersen TL, Garvey WT, Newman JW (2012). Type 2 diabetes associated changes in the plasma non-esterified fatty acids, oxylipins and endocannabinoids. PLoS ONE.

[15] Picklo MJ, Newman JW (2015). Antioxidant supplementation and obesity have independent effects on hepatic oxylipin profiles in insulin-resistant, obesity-prone rats. Free Radic. Biol. Med..

[16] Gabbs M, Leng S, Devassy JG, Monirujjaman M, Aukema HM (2015). Advances in our understanding of oxylipins derived from dietary PUFAs. Adv. Nutr..

[17] Jones RD (2019). Epoxy-oxylipins and soluble epoxide hydrolase metabolic pathway as targets for NSAID-induced gastroenteropathy and inflammation-associated carcinogenesis. Front. Pharmacol..

[18] Nayeem MA (2018). Role of oxylipins in cardiovascular diseases. Acta Pharmacol. Sin..

[19] Barquissau V (2017). Control of adipogenesis by oxylipins, GPCRs and PPARs. Biochimie.

[20] Ahmad TR, Haeusler RA (2019). Bile acids in glucose metabolism and insulin signalling: Mechanisms and research needs. Nat. Rev. Endocrinol..

[21] Li T, Apte U (2015). Bile acid metabolism and signaling in cholestasis, inflammation, and cancer. Adv. Pharmacol..

[22] Chiang JY (2013). Bile acid metabolism and signaling. Compr. Physiol..

[23] Ferrell JM, Chiang JYL (2019). Understanding bile acid signaling in diabetes: From pathophysiology to therapeutic targets. Diabetes Metab. J..

[24] Devassy JG, Leng S, Gabbs M, Monirujjaman M, Aukema HM (2016). Omega-3 polyunsaturated fatty acids and oxylipins in neuroinflammation and management of Alzheimer disease. Adv Nutr.

[25] Nho K (2019). Altered bile acid profile in mild cognitive impairment and Alzheimer's disease: Relationship to neuroimaging and CSF biomarkers. Alzheimers Dementia.

[26] MahmoudianDehkordi S (2019). Altered bile acid profile associates with cognitive impairment in Alzheimer's disease: An emerging role for gut microbiome. Alzheimers Dementia.

[27] Vest RS, Pike CJ (2013). Gender, sex steroid hormones, and Alzheimer's disease. Horm. Behav..

[28] Lv W (2016). Low testosterone level and risk of Alzheimer's disease in the elderly men: A systematic review and meta-analysis. Mol. Neurobiol..

[29] Yu D (2018). Soluble epoxide hydrolase-derived linoleic acid oxylipins in serum are associated with periventricular white matter hyperintensities and vascular cognitive impairment. Transl. Stroke Res..

[30] Bennett DA (2018). Religious orders study and rush memory and aging project. J. Alzheimers Dis..

[31] Folstein MF, Folstein SE, McHugh PR (1975). "Mini-mental state". A practical method for grading the cognitive state of patients for the clinician. J. Psychiatr. Res..

[32] It D (1973). The assessment of aphasia and related disorders. J. Neurol. Neurosurg. Psychiatry.

[33] Welsh KA, Butters N, Hughes JP, Mohs RC, Heyman A (1992). Detection and staging of dementia in Alzheimer's disease Use of the neuropsychological measures developed for the Consortium to Establish a Registry for Alzheimer's Disease. Arch. Neurol..

[34] Albert M (1991). Use of brief cognitive tests to identify individuals in the community with clinically diagnosed Alzheimer's disease. Int. J. Neurosci..

[35] D., W. *Wechsler Memory Scale-Revised manual.* (1987).

[36] Kaplan, E. F., Goodglass, H., & Weintraub, S. The Boston Naming Test. Philadelphia: Lea & Febiger. *The Boston Naming Test*. (1983).

[37] Lezak M., H. D., Bigler E., Tranel D. *Neuropsychological Assessment.* (2012).

[38] Grober E, Sliwinski M (1991). Development and validation of a model for estimating premorbid verbal intelligence in the elderly. J. Clin. Exp. Neuropsychol..

[39] Blair JR, Spreen O (1989). Predicting premorbid IQ: A revision of the National Adult Reading Test. Clin. Neuropsychol..

[40] Nelson, H. E. *National Adult Reading Test (NART): Test manual.* (1982).

[41] Cooper JA, Sagar HJ, Jordan N, Harvey NS, Sullivan EV (1991). Cognitive impairment in early, untreated Parkinson's disease and its relationship to motor disability. Brain.

[42] Cooper JA (1992). Different effects of dopaminergic and anticholinergic therapies on cognitive and motor function in Parkinson's disease. A follow-up study of untreated patients. Brain.

[43] Cooper JA, Sagar HJ (1993). Incidental and intentional recall in Parkinson's disease: An account based on diminished attentional resources. J. Clin. Exp. Neuropsychol..

[44] A., S. *Symbol Digit Modalities Test manual-revised* (Western Psychological, 1984).

[45] Ekstrom RB, F. J., Harman HH, Dermen D. *Manual for kit of factor-referenced cognitive tests *(Educational Testing Service, 1976).

[46] Benton AL, Varney NR, Hamsher KD (1978). Visuospatial judgment. A clinical test. Arch. Neurol..

[47] Raven JC, C. J., Raven J. *Standard progressive matrices-1992 edition; Raven manual:Section 3**, *(Oxford Psychologists Press, 1992).

[48] Bennett DA (2006). Decision rules guiding the clinical diagnosis of Alzheimer's disease in two community-based cohort studies compared to standard practice in a clinic-based cohort study. Neuroepidemiology.

[49] Bennett DA (2002). Natural history of mild cognitive impairment in older persons. Neurology.

[50] Bennett DA (2006). Neuropathology of older persons without cognitive impairment from two community-based studies. Neurology.

[51] Lamar M (2019). Associations of literacy with diabetes indicators in older adults. J. Epidemiol. Community Health.

[52] La Frano MR (2017). Diet-induced obesity and weight loss alter bile acid concentrations and bile acid-sensitive gene expression in insulin target tissues of C57BL/6J mice. Nutr. Res..

[53] Pedersen TL, Newman JW (2018). Establishing and performing targeted multi-residue analysis for lipid mediators and fatty acids in small clinical plasma samples. Methods Mol. Biol.

[54] Benjamini Y, Yekutieli D (2005). Quantitative trait loci analysis using the false discovery rate. Genetics.

[55] Goetz ME (2019). Rationale and design of the emory healthy aging and emory healthy brain studies. Neuroepidemiology.

[56] Lee CR (2006). Genetic variation in soluble epoxide hydrolase (EPHX2) and risk of coronary heart disease: The Atherosclerosis Risk in Communities (ARIC) study. Hum. Mol. Genet..

[57] O'Brien JT, Thomas A (2015). Vascular dementia. Lancet.

[58] Raz L, Knoefel J, Bhaskar K (2016). The neuropathology and cerebrovascular mechanisms of dementia. J. Cereb. Blood Flow Metab..

[59] Schneider JA, Arvanitakis Z, Leurgans SE, Bennett DA (2009). The neuropathology of probable Alzheimer disease and mild cognitive impairment. Ann Neurol.

[60] Racz B (2015). Daily profiles of steroid hormones and their metabolites related to food intake. Physiol Res.

[61] Camargo A (2013). Postprandial changes in the proteome are modulated by dietary fat in patients with metabolic syndrome. J. Nutr. Biochem..

[62] Sagaya FM, Hurrell RF, Vergeres G (2012). Postprandial blood cell transcriptomics in response to the ingestion of dairy products by healthy individuals. J. Nutr. Biochem..

[63] Zheng M (2019). Relationship between inflammatory markers and mild cognitive impairment in Chinese patients with type 2 diabetes: A case-control study. BMC Endocr. Disord..

[64] Chu L, Morrison KM, Riddell MC, Raha S, Timmons BW (2018). Validity and reliability of a novel metabolic flexibility test in children with obesity. J. Appl. Physiol..

[65] Kumar AA (2020). Postprandial metabolism is impaired in overweight normoglycemic young adults without family history of diabetes. Sci. Rep..

[66] de Vries MA (2014). Postprandial inflammation: Targeting glucose and lipids. Adv. Exp. Med. Biol..

[67] La Frano MR (2018). Impact of post-collection freezing delay on the reliability of serum metabolomics in samples reflecting the California mid-term pregnancy biobank. Metabolomics.

[68] Koch E (2020). Stability of oxylipins during plasma generation and long-term storage. Talanta.

[69] Karpe F, Steiner G, Uffelman K, Olivecrona T, Hamsten A (1994). Postprandial lipoproteins and progression of coronary atherosclerosis. Atherosclerosis.

[70] Jackson KG, Wolstencroft EJ, Bateman PA, Yaqoob P, Williams CM (2005). Acute effects of meal fatty acids on postprandial NEFA, glucose and apo E response: Implications for insulin sensitivity and lipoprotein regulation?. Br. J. Nutr/.

[71] Snowden SG (2017). Association between fatty acid metabolism in the brain and Alzheimer disease neuropathology and cognitive performance: A nontargeted metabolomic study. PLoS Med.

[72] Baierle M (2014). Fatty acid status and its relationship to cognitive decline and homocysteine levels in the elderly. Nutrients.

[73] Cunnane SC (2012). Plasma and brain fatty acid profiles in mild cognitive impairment and Alzheimer's disease. J. Alzheimers Dis..

[74] Turcotte C, Chouinard F, Lefebvre JS, Flamand N (2015). Regulation of inflammation by cannabinoids, the endocannabinoids 2-arachidonoyl-glycerol and arachidonoyl-ethanolamide, and their metabolites. J. Leukoc. Biol..

[75] Petrosino S, Di Marzo V (2017). The pharmacology of palmitoylethanolamide and first data on the therapeutic efficacy of some of its new formulations. Br. J. Pharmacol..

[76] Joosten MM, Balvers MG, Verhoeckx KC, Hendriks HF, Witkamp RF (2010). Plasma anandamide and other N-acylethanolamines are correlated with their corresponding free fatty acid levels under both fasting and non-fasting conditions in women. Nutr. Metab. (Lond.).

[77] Bradshaw HB, Walker JM (2005). The expanding field of cannabimimetic and related lipid mediators. Br. J. Pharmacol..

[78] Saito VM, Rezende RM, Teixeira AL (2012). Cannabinoid modulation of neuroinflammatory disorders. Curr. Neuropharmacol..

[79] Bradshaw HB, Raboune S, Hollis JL (2013). Opportunistic activation of TRP receptors by endogenous lipids: Exploiting lipidomics to understand TRP receptor cellular communication. Life Sci..

[80] Kim SR (2007). Roles of transient receptor potential vanilloid subtype 1 and cannabinoid type 1 receptors in the brain: Neuroprotection versus neurotoxicity. Mol. Neurobiol..

[81] Ho M, Anderson GH, Lin L, Bazinet RP, Kubant R (2020). Ethanolamides of essential alpha-linolenic and linoleic fatty acids suppress short-term food intake in rats. Food Funct..

[82] Frigolet ME, Gutierrez-Aguilar R (2017). The role of the novel lipokine palmitoleic acid in health and disease. Adv. Nutr..

[83] MahmoudianDehkordi S (2019). Altered bile acid profile associates with cognitive impairment in Alzheimer's disease: An emerging role for gut microbiome. Alzheimers Dement..

[84] Nho K (2019). Altered bile acid profile in mild cognitive impairment and Alzheimer's disease: Relationship to neuroimaging and CSF biomarkers. Alzheimers Dement..

[85] Wopereis S (2017). Multi-parameter comparison of a standardized mixed meal tolerance test in healthy and type 2 diabetic subjects: The PhenFlex challenge. Genes Nutr..

[86] Castro Cabezas M, Botham KM, Mamo JC, Proctor SD (2012). Novel aspects of nonfasting lipemia in relation to vascular biology. Int. J. Vasc. Med..

[87] Rajamani A (2019). Oxylipins in triglyceride-rich lipoproteins of dyslipidemic subjects promote endothelial inflammation following a high fat meal. Sci. Rep..

[88] Toth P, Tarantini S, Csiszar A, Ungvari Z (2017). Functional vascular contributions to cognitive impairment and dementia: Mechanisms and consequences of cerebral autoregulatory dysfunction, endothelial impairment, and neurovascular uncoupling in aging. Am. J. Physiol. Heart Circ. Physiol..

[89] Yu D (2019). Soluble epoxide hydrolase-derived linoleic acid oxylipins in serum are associated with periventricular white matter hyperintensities and vascular cognitive impairment. Transl. Stroke Res..

[90] Shinto L (2020). Oxidized products of omega-6 and omega-3 long chain fatty acids are associated with increased white matter hyperintensity and poorer executive function performance in a cohort of cognitively normal hypertensive older adults. J. Alzheimers Dis..

[91] Wagner KM, McReynolds CB, Schmidt WK, Hammock BD (2017). Soluble epoxide hydrolase as a therapeutic target for pain, inflammatory and neurodegenerative diseases. Pharmacol. Ther..

[92] Hashimoto K (2019). Role of soluble epoxide hydrolase in metabolism of PUFAs in psychiatric and neurological disorders. Front. Pharmacol..

[93] Pardeshi R (2019). Docosahexaenoic acid increases the potency of soluble epoxide hydrolase inhibitor in alleviating streptozotocin-induced Alzheimer's disease-like complications of diabetes. Front. Pharmacol..

[94] Bell SM (2018). Ursodeoxycholic acid improves mitochondrial function and redistributes Drp1 in fibroblasts from patients with either sporadic or familial Alzheimer's disease. J. Mol. Biol..

[95] Keene CD (2002). Tauroursodeoxycholic acid, a bile acid, is neuroprotective in a transgenic animal model of Huntington's disease. Proc. Natl. Acad. Sci. USA.

[96] Sola S, Castro RE, Laires PA, Steer CJ, Rodrigues CM (2003). Tauroursodeoxycholic acid prevents amyloid-beta peptide-induced neuronal death via a phosphatidylinositol 3-kinase-dependent signaling pathway. Mol. Med..

[97] Ruan Y (2019). Mixed-meal tolerance test to assess residual beta-cell secretion: Beyond the area-under-curve of plasma C-peptide concentration. Pediatr. Diabetes.

