# Supplementary material for: Serum metabolomic biomarkers of perceptual speed in cognitively normal and mildly impaired subjects with fasting state stratification
Source: Sci Rep. 2021 Sep 23;11:18964. doi: 10.1038/s41598-021-98640-2 (PMC8460824; doi:10.1038/s41598-021-98640-2)
Supplement: Supplementary file 1 — Supplementary Information 1. [file 41598_2021_98640_MOESM1_ESM.pdf]

Title: Serum metabolomic biomarkers of perceptual speed in cognitively normal and mildly impaired subjects with fasting state stratification

Authors: Kamil Borkowski, Ameer Y. Taha, Theresa L. Pedersen, Philip L. De Jager, David A. Bennett, Rima Kaddurah-Daouk, John W. Newman

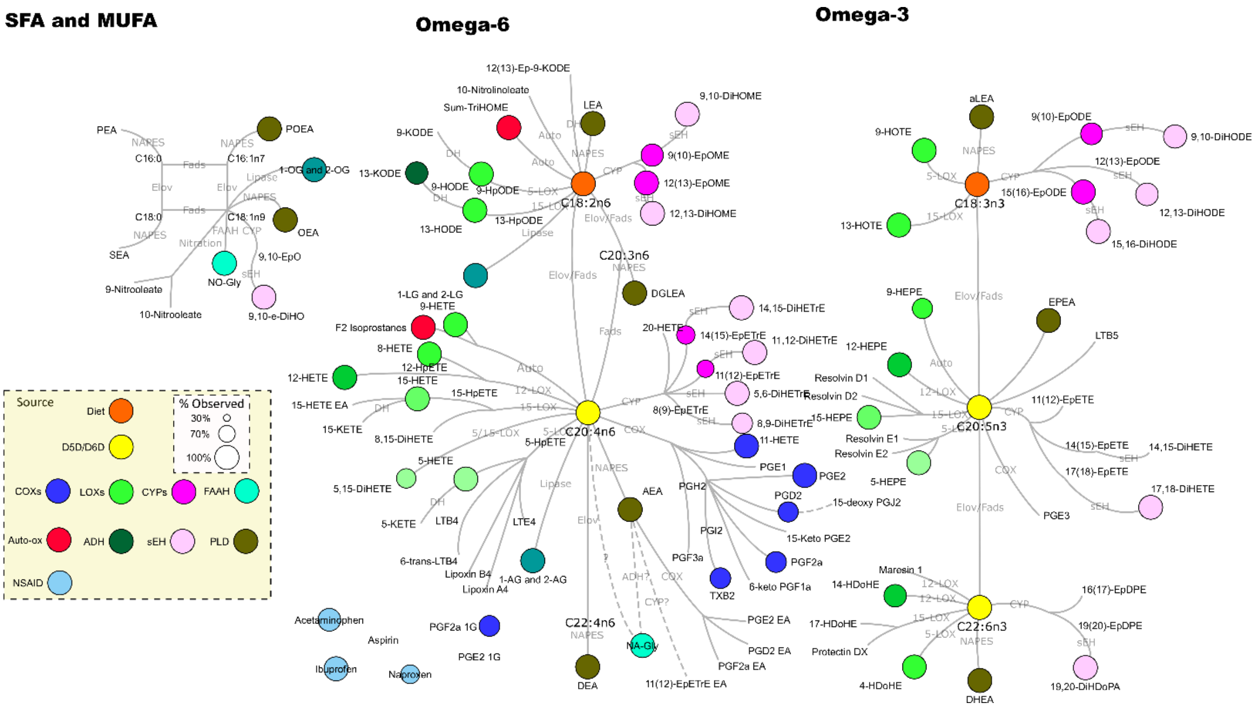

Supplemental Figure S1. Oxylipins, endocannabinoids, fatty acids and NSAIDs detected in serum of experimental cohort. Detected metabolites are presented as colored node. Node color represents metabolic pathway/source of metabolite whereas size represents detection frequency. Edge label describe metabolizing enzyme. D5D/D6D – delta 5 and delta 6 desaturase, COXs – cyclooxygenases, LOXs, lipoxygenases, CYPs – cytochrome p450, FAAH – fatty acid amide hydrolase, ADH – alcohol dehydrogenase, sEH – soluble epoxide hydrolase, PLD – phospholipase D, NSAIDs – non-steroidal anti-inflammatory drugs.
