# Supplementary material for: Serum metabolomic biomarkers of perceptual speed in cognitively normal and mildly impaired subjects with fasting state stratification
Source: Sci Rep. 2021 Sep 23;11:18964. doi: 10.1038/s41598-021-98640-2 (PMC8460824; doi:10.1038/s41598-021-98640-2)
Supplement: Supplementary file 2 — Supplementary Information 2. [file 41598_2021_98640_MOESM2_ESM.pdf]

Title: Serum metabolomic biomarkers of perceptual speed in cognitively normal and mildly impaired subjects with fasting state stratification

Authors: Kamil Borkowski, Ameer Y. Taha, Theresa L. Pedersen, Philip L. De Jager, David A. Bennett, Rima Kaddurah-Daouk, John W. Newman

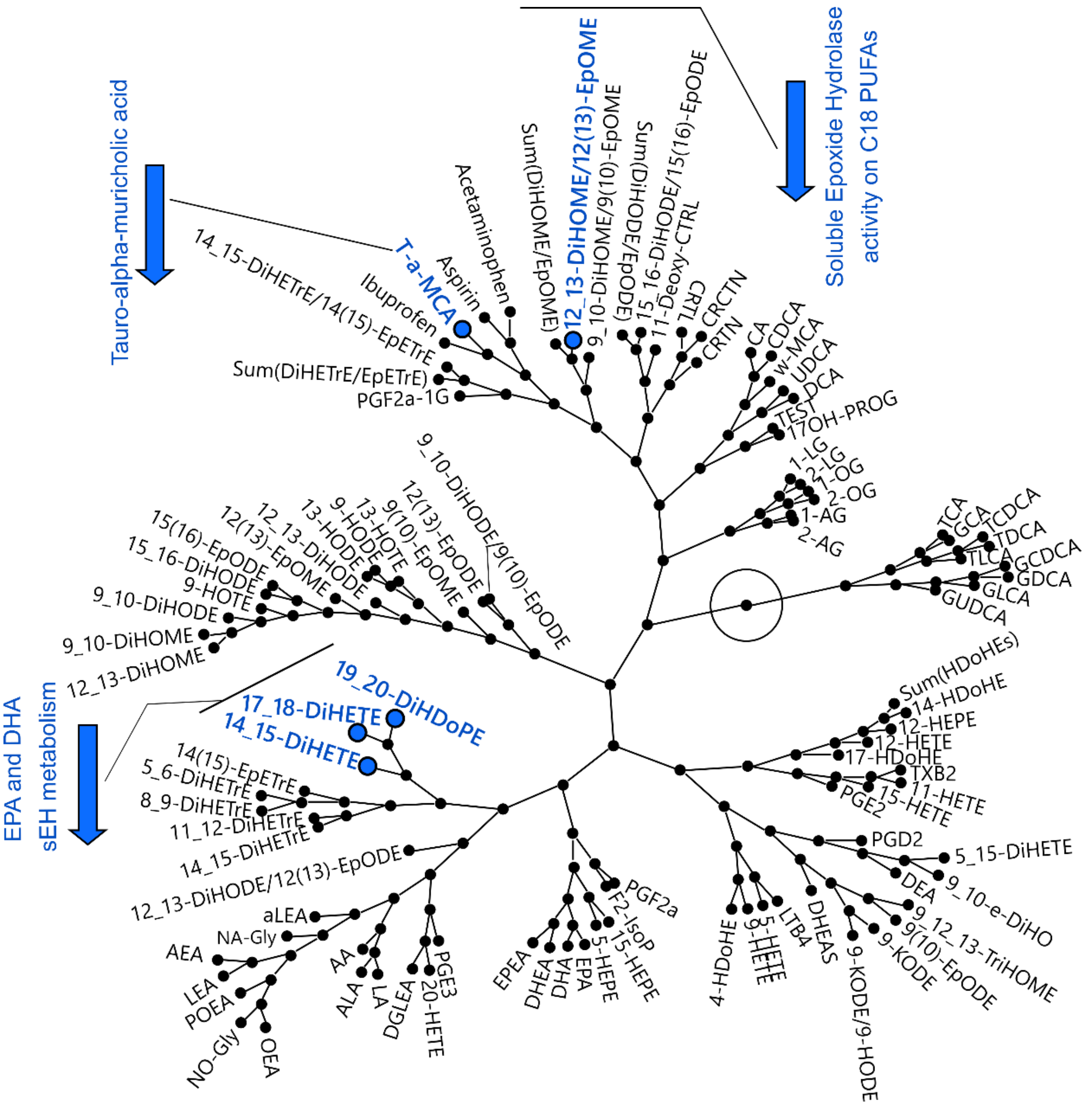

Supplemental Figure S2. Constituent metabolites of perceptual speed predictive model, projected onto metabolite correlation network. Constellation plot of hierarchical cluster analysis of oxylipins, endocannabinoids, PUFA and bile acids, recorded in the fasted samples.

Metabolites used un the model are highlighted. Colors indicate directionality of correlation with perceptual speed (blue – negative). General description is provided for metabolites closely correlated with those used in the predictive model.
