# Supplementary material for: Serum metabolomic biomarkers of perceptual speed in cognitively normal and mildly impaired subjects with fasting state stratification
Source: Sci Rep. 2021 Sep 23;11:18964. doi: 10.1038/s41598-021-98640-2 (PMC8460824; doi:10.1038/s41598-021-98640-2)
Supplement: Supplementary file 4 — Supplementary Information 4. [file 41598_2021_98640_MOESM4_ESM.pdf]

Title: Serum metabolomic biomarkers of perceptual speed in cognitively normal and mildly impaired subjects with fasting state stratification

Authors: Kamil Borkowski, Ameer Y. Taha, Theresa L. Pedersen, Philip L. De Jager, David A. Bennett, Rima Kaddurah- Daouk, John W. Newman

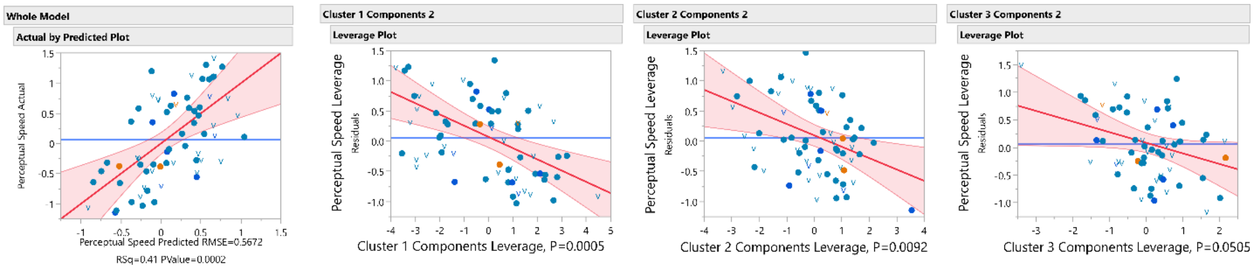

Effect Summary

Source	LogWorth	PValue
Cluster 1 Components	3.316	0.00048
Cluster 2 Components	2.037	0.00919
Cluster 3 Components	1.296	0.05055

Crossvalidation

Source	RSquare	RASE	Freq
Training Set	0.4050	0.53948	42
Validation Set	0.2934	0.58339	26

Supplemental Figure S4. Perceptual speed predictive model build using cluster component of variables preselected for the model using bootstrap forest.
