# Supplementary material for: Serum metabolomic biomarkers of perceptual speed in cognitively normal and mildly impaired subjects with fasting state stratification
Source: Sci Rep. 2021 Sep 23;11:18964. doi: 10.1038/s41598-021-98640-2 (PMC8460824; doi:10.1038/s41598-021-98640-2)
Supplement: Supplementary file 5 — Supplementary Information 5. [file 41598_2021_98640_MOESM5_ESM.pdf]

Title: Serum metabolomic biomarkers of perceptual speed in cognitively normal and mildly impaired subjects with fasting state stratification

Authors: Kamil Borkowski, Ameer Y. Taha, Theresa L. Pedersen, Philip L. De Jager, David A. Bennett, Rima Kaddurah-Daouk, John W. Newman

Supplemental Table S1. Means and 95% CI for the global cognition, cognitive domains and cognitive tests used to generate cognitive domain scores. NCI - no cognitive impairment; MCI - mild cognitive impairment, no other condition contributing to cognitive impairment.

	Female				Male			
	Fasting		Non-Fasting		Fasting		Non-Fasting	
	NCI (n=48)	MCI (n=6)	NCI (n=89)	MCI (n=21)	NCI (n=13)	MCI (n=4)	NCI (n=27)	MCI (n=4)
Global cognition (mean ± 95%CI)	0.341 ± 0.126	-0.00117 ± 0.478	0.351 ± 0.0816	-0.204 ± 0.162	0.514 ± 0.234	-0.456 ± 0.529	0.229 ± 0.177	-0.423 ± 0.736
Cognitive domines (mean ± 95%CI)								
Episodic memory	0.427 ± 0.163	0.0886 ± 0.748	0.411 ± 0.0968	-0.397 ± 0.264	0.432 ± 0.298	-0.816 ± 0.79	0.238 ± 0.175	-0.944 ± 0.56
Perceptual orientation	0.367 ± 0.197	-0.455 ± 0.581	0.29 ± 0.143	-0.375 ± 0.361	0.857 ± 0.327	0.675 ± 1.21	0.57 ± 0.261	0.598 ± 1.69
Perceptual speed	0.33 ± 0.21	0.134 ± 0.732	0.314 ± 0.147	-0.129 ± 0.352	0.835 ± 0.47	-0.481 ± 1.29	0.262 ± 0.305	-0.301 ± 1.78
Sementiac memory	0.269 ± 0.16	0.31 ± 0.476	0.344 ± 0.106	-0.0605 ± 0.277	0.338 ± 0.27	-0.513 ± 0.284	0.177 ± 0.273	-0.0983 ± 0.93
Working memory	0.194 ± 0.206	-0.309 ± 0.69	0.264 ± 0.139	0.166 ± 0.229	0.457 ± 0.228	-0.296 ± 0.492	-0.0063 ± 0.25	-0.295 ± 0.609
Tests used for episodic memory (mean ± 95%CI)								
Logical memory II (delayed recall)	11.4 ± 1.21	8.57 ± 5.47	11.8 ± 0.77	7.24 ± 2.14	12.5 ± 2.25	4.5 ± 4.21	10 ± 1.37	2 ± 3.9
Logical memory I (immediate recall)	13 ± 1.14	12 ± 4.2	13.3 ± 0.696	8.95 ± 1.97	14.2 ± 2.08	8 ± 5.03	11.5 ± 1.24	5 ± 2.25
word list	20 ± 1.25	17.1 ± 5.59	19.2 ± 0.826	15.9 ± 1.73	17.7 ± 2.26	15.3 ± 7.16	17.8 ± 1.44	14 ± 2.91
word list recall	6.84 ± 0.566	5.57 ± 2.82	6.46 ± 0.384	3.52 ± 1.09	6.67 ± 0.828	3 ± 2.25	5.76 ± 0.632	3.75 ± 2.72
word list recognition	9.86 ± 0.0977	9.57 ± 0.728	9.93 ± 0.053	8.95 ± 0.741	9.67 ± 0.342	8.75 ± 2.39	9.83 ± 0.146	9 ± 1.84
East Boston delayed recall	9.8 ± 0.531	9.86 ± 1.72	9.81 ± 0.343	8 ± 1.38	9.87 ± 1.14	6.25 ± 2.39	9.66 ± 0.971	6.5 ± 3.79
East Boston immediate recall	9.96 ± 0.506	9.43 ± 1.99	10 ± 0.327	9 ± 0.814	10.3 ± 1.12	7 ± 1.3	10.6 ± 0.637	7 ± 2.91
Tests used for perceptual orientiation (mean ± 95%CI)								
progressive matrices (16 items)	13 ± 0.663	10.1 ± 3.18	12.7 ± 0.499	9.95 ± 1.42	13.6 ± 1.28	13 ± 5.66	13.3 ± 0.848	13 ± 2.91
line orientation	10.3 ± 0.923	7.86 ± 2.29	10.2 ± 0.584	8.57 ± 1.38	12.7 ± 1.14	12.3 ± 2.39	11.4 ± 1.27	11.8 ± 8.36
Tests used for perceptual speed (mean ± 95%CI)								
number comparison	26.9 ± 2.06	26.6 ± 5.94	26.4 ± 1.42	23.5 ± 2.84	31.5 ± 4.77	22 ± 4.68	26.1 ± 2.5	22.5 ± 12.6
symbol digits modality test (oral)	42.5 ± 2.37	38.3 ± 9.94	42.8 ± 1.71	36.8 ± 4.58	47.3 ± 4.5	30.8 ± 27.6	42.1 ± 4.16	34.3 ± 24.1
stroop color naming	20.6 ± 1.78	21 ± 4.89	21.2 ± 1.27	16.4 ± 3.46	22.5 ± 4.64	11.8 ± 7.16	20.4 ± 2.68	18.8 ± 17.7
stroop word reading	49.2 ± 4.01	53.1 ± 12.8	52.3 ± 2.46	46.9 ± 7.13	56.3 ± 7.53	41.8 ± 6.15	50.9 ± 4.84	51.8 ± 33.2
Tests used for sementic memory (mean ± 95%CI)								
reading test - (10 items)	8.02 ± 0.58	7.71 ± 2.25	8.47 ± 0.395	8.19 ± 0.797	8.53 ± 0.779	5.75 ± 1.52	7.76 ± 1.04	7.75 ± 3.28
Boston naming (15 items)	14.4 ± 0.244	14.3 ± 0.699	14.3 ± 0.179	13.4 ± 0.669	14.3 ± 0.442	14 ± 1.3	14.5 ± 0.262	14.3 ± 1.52
CatFlu category fluency (animals - fruits/vegetables)	37.4 ± 2.45	40.3 ± 5.31	38.3 ± 1.68	32.4 ± 3.57	37.7 ± 5.67	26 ± 10.3	34.8 ± 4.39	28.3 ± 14.3
Tests used for working memory (mean ± 95%CI)								
digits backward	6.37 ± 0.614	5.43 ± 1.76	6.75 ± 0.401	6.1 ± 0.8	6.47 ± 0.909	5 ± 2.25	5.52 ± 0.581	3.75 ± 1.52
digits forward	8.47 ± 0.529	7.14 ± 1.72	8.57 ± 0.411	8.62 ± 0.667	9.2 ± 0.789	8 ± 3.44	8.17 ± 0.719	8.5 ± 2.05
digit ordering	7.76 ± 0.428	6.86 ± 1.46	7.68 ± 0.262	7.67 ± 0.485	8.53 ± 0.623	6.5 ± 0.919	7.62 ± 0.678	7.25 ± 0.796
Biometrics (mean ± SD)								
Age	76.7 ± 7.95	80.2 ± 7.07	78.4 ± 7.39	79.8 ± 5.05	78.3 ± 5.79	84.3 ± 6.44	79.6 ± 6.98	79.5 ± 10.9
BMI	27.8 ± 4.73	30 ± 4.98	26.8 ± 4.75	25.4 ± 3.59	30 ± 5.02	23.5 ± 2.76	27.6 ± 4.57	27.1 ± 5.18
Education	14.8 ± 2.46	16.7 ± 2.5	15.2 ± 2.62	15.5 ± 2.27	15.6 ± 2.97	18 ± 2.16	15.6 ± 3.52	15.8 ± 4.19
Race								
White Caucasians	94%	100%	97%	90%	100%	100%	100%	75%
African American	4%	-	3%	5%	-	-	-	25%
Other	2%	-		5%				
