# Supplementary material for: Serum metabolomic biomarkers of perceptual speed in cognitively normal and mildly impaired subjects with fasting state stratification
Source: Sci Rep. 2021 Sep 23;11:18964. doi: 10.1038/s41598-021-98640-2 (PMC8460824; doi:10.1038/s41598-021-98640-2)
Supplement: Supplementary file 6 — Supplementary Information 6. [file 41598_2021_98640_MOESM6_ESM.pdf]

Title: Serum metabolomic biomarkers of perceptual speed in cognitively normal and mildly impaired subjects with fasting state stratification

Authors: Kamil Borkowski, Ameer Y. Taha, Theresa L. Pedersen, Philip L. De Jager, David A. Bennett, Rima Kaddurah-Daouk, John W. Newman

Supplemental Table S2. Oxylipins, endocannabinoids, PUFAs, NSAIDs, bile acids and steroids specific information and identifiers.

Report Order	Analyte-ID	LOD (NM)	LOQ (NM)	Observed	InChIKey	PubChem CID	Chemical class	Enzyme	Parent FA
					**Oxylipins**				
1	TXB2	0.233	0.699	Yes	XNRNNGPBEPRNAR-JQBLCGNGSA-N	5283137	TX	COX1	AA
2	6-keto-PGF1a	0.253	0.759	No	KFGOFTHODYBSGM-ZUNNJUQCSA-N	5280888	PGs	COX1	AA
3	PGE1	0.568	1.7	No	GMVPRGQOIOIIMI-DWKJAMRDSA-N	5280723	PGs	COX2	DGLA
4	PGE2	0.198	0.595	Yes	XEYBRNLFEZDVAW-ARSRFYASSA-N	5280360	PGs	COX2	AA
5	15-Keto-PGE2	0.287	0.862	No	YRTJDWROBKPZNV-KMXMBPPJSA-N	5280719	PGs	COX2	AA
6	PGD2	0.267	0.802	Yes	BHMBVRSPMRCCGG-OUTUXVNYSA-N	448457	PGs	COX2	AA
7	15-deoxy-PGJ2	0.0824	0.247	No	VHRUMKCAEVRUBK-GODQJPCRSA-N	5311211	PGs	COX2	AA
8	PGF2a	0.299	0.897	Yes	PXGPLTODNUVGFL-UAAPODJFSA-N	5283078	PGs	COX2	AA
9	PGE3	0.37	1.11	Yes	CBOMORHDRONZRN-QLOYDKTKSA-N	5280937	PGs	COX2	EPA
10	PGF3a	0.369	1.11	No	SAKGBZWJAIABSY-SAMSIYEGSA-N	5280940	PGs	COX2	EPA
11	F2-Isoprostanes	0.243	0.729	Yes	---	---	PGs	Auto-ox	AA
12	PGE2-EA	0.264	0.792	No	GKKWUSPPIQURFM-IGDGGSTLSA-N	5283119	PG-EA	COX2	AA
13	PGF2a-EA	0.29	0.871	Yes	XCVCLIRZZCGEMU-FPLRWIMGSA-N	53481911	PG-EA	COX2	AA
14	PGD2-EA	0.484	1.45	No	KEYDJKSQFDUAGF-YIRKRNQHSA-N	5283120	PG-EA	COX2	AA
15	PGF2a-1G	0.468	1.4	Yes	NWKPOVHSHWJQNI-OMVDPNNKSA-N	24778485	PG-Gly	COX2	AA
16	PGE2-1G	0.645	1.93	No	RJXVYMMSQBYEHN-SDTVLRMPSA-N	52193688	PG-Gly	COX2	AA
17	LTE4			No	OTZRAYGBFWZKMX-MPWKMEBCSA-N	5280749	Cys-LT	sEH	AA
18	6-trans-LTB4	0.99	2.97	No	VNYSSYRCGWBHLG-UKNWISKWSA-N	5283128	Diol	LOX	AA
19	LTB4	0.217	0.652	Yes	KFGOFTHODYBSGM-ZUNNJUQCSA-N	5280888	Diol	LOX	AA
20	LTB5	0.0955	0.286	No	BISQPGCQOHLHQK-HDNPQISLSA-N	5283125	Diol	LOX	AA
21	8_15-DiHETE	0.614	1.84	No	NNPWRKSGORGTIM-RCDCWWQHSA-N	53480358	Diol	LOX	AA
22	5_15-DiHETE	0.352	1.06	Yes	UXGXCGPWGSUMNI-BVHTXILBSA-N	5283158	Diol	LOX	AA
23	9_12_13-TriHOME	0.276	0.828	Yes	MDIUMSLCYIJBQC-MVFSOIOZSA-N	9858729	Triol	LOX	LA
24	Lipoxin-B4	0.521	1.56	No	UXVRTOKOJOMENI-WLPVFMORSA-N	5280915	Triol	LOX	AA
25	Resolvin-E1-Screen			No	AOPOCGPBAIARAV-WEKRNNBPSA-N	25063347	Triol	LOX	AA
26	Lipoxin-A4	0.376	1.13	No	IXAQOQZEOGMIQS-SSQFXEBMSA-N	5280914	Triol	LOX	AA
27	Resolvin-E2-Screen			No	KPRHYAOSTOHNQA-NNQKPOSRSA-N	16061125	Triol	LOX	AA
28	Resolvin-D1	0.228	0.685	No	OIWTWACQMDFHJG-NJIQAZPPSA-N	16061135	Triol	LOX	DHA
29	Resolvin-D2	0.704	2.11	No	IKFAUGXNBOBQDM-XFMPMKITSA-N	11383310	Triol	LOX	DHA
30	Protectin-DX	0.186	0.558	No	CRDZYJSQHCXHEG-XLBFCUQGSA-N	11667655	Diol	LOX	DHA
31	Maresin-1	0.921	2.76	No	HLHYXXBCQOUTGK-LUSCUACYSA-N	102518309	Triol	LOX	DHA
32	9_10-e-DiHO	1.15	3.45	Yes	VACHUYIREGFMSP-SJORKVTESA-N	441460	vic-Diol	sEH	OA
33	12_13-DiHOME	0.212	0.635	Yes	CQSLTKIXAJTQGA-FLIBITNWSA-N	10236635	vic-Diol	sEH	LA

Continuation of **Table S1**.

Report Order	Analyte-ID	LOD (NM)	LOQ (NM)	Observed	InChIKey	PubChem CID	Chemical class	Enzyme	Parent FA
34	9_10-DiHOME	0.206	0.617	Yes	XEBKSQSGNGRGDW-YFHOEESVSA-N	9966640	vic-Diol	sEH	LA
35	15_16-DiHODE	0.311	0.934	Yes	LKLLJYJTYPVCID-OHPMOLHNSA-N	16061068	vic-Diol	sEH	aLA
36	12_13-DiHODE	0.187	0.561	Yes	RGRKFKRAFZJQMS-OOHFSOINSA-N	16061067	vic-Diol	sEH	aLA
37	9_10-DiHODE	0.32	0.961	Yes	QRHSEDZBZMZPOA-ZJSQCTGTSA-N	16061066	vic-Diol	sEH	aLA
38	14_15-DiHETrE	0.172	0.515	Yes	SYAWGTIVOGUZMM-ILYOTBPNSA-N	5283147	vic-Diol	sEH	AA
39	11_12-DiHETrE	0.112	0.336	Yes	LRPPQRCHCPFBPE-KROJNAHFSA-N	5283146	vic-Diol	sEH	AA
40	8_9-DiHETrE	0.298	0.895	Yes	DCJBINATHQHPKO-TYAUOURKSA-N	5283144	vic-Diol	sEH	AA
41	5_6-DiHETrE	0.136	0.407	Yes	GFNYAPAJUNPMGH-QNEBEIHSSA-N	5283142	vic-Diol	sEH	AA
42	14_15-DiHETE	0.998	3	Yes	BLWCDFIELVFRJY-QXBXTPPVSA-N	16061119	vic-Diol	sEH	EPA
43	17_18-DiHETE	1.08	3.24	Yes	XYDVGNAQQFWZEF-JPURVOHMSA-N	16061120	vic-Diol	sEH	EPA
44	19_20-DiHDoPE	0.211	0.632	Yes	FFXKPSNQCPNORO-MBYQGORISA-N	16061148	vic-Diol	sEH	DHA
45	13-HODE	1.18	3.55	Yes	HNICUWMFWZBIFP-IRQZEAMPSA-N	6443013	R-OH	LOX	LA
46	9-HODE	0.645	1.94	Yes	NPDSHTNEKLQQIJ-SIGMCMEVSA-N	5282945	R-OH	LOX	LA
47	13-HOTE	0.405	1.22	Yes	KLLGGGQNRTVBSU-JDTPQGGVSA-N	10469728	R-OH	LOX	aLA
48	9-HOTE	0.207	0.621	Yes	YUPHIKSLGBATJK-OBKPXJAFSA-N	53480359	R-OH	LOX	aLA
49	20-HETE	0.397	1.19	Yes	NNDIXBJHNLFJJP-DTLRTWKJSA-N	5283157	R-OH	CYP	AA
50	15-HETE	0.208	0.625	Yes	JSFATNQSLKRBCI-VAEKSGALSA-N	5280724	R-OH	LOX	AA
51	12-HETE	0.173	0.518	Yes	ZNHVWPKMFKADKW-FYMOKONMSA-N	5312983	R-OH	LOX	AA
52	11-HETE	0.25	0.749	Yes	GCZRCCHPLVMMJE-RSPKXIRXSA-N	5312981	R-OH	LOX	AA
53	9-HETE	0.63	1.89	Yes	KATOYYZUTNAWSA-DLJQHUEDSA-N	5312978	R-OH	LOX	AA
54	8-HETE	0.651	1.95	Yes	NLUNAYAEIJYXRB-VYOQERLCSA-N	5283154	R-OH	LOX	AA
55	5-HETE	0.242	0.726	Yes	KGIJOOYOSFUGPC-JGKLHWIESA-N	5280733	R-OH	LOX	AA
56	15-HEPE	0.191	0.574	Yes	UDXLGBLAJBYLSZ-XBCQTNLFSA-N	53480357	R-OH	LOX	EPA
57	12-HEPE	0.312	0.935	Yes	MCRJLMXYVFDXLS-QGQBRVLBSA-N	10041593	R-OH	LOX	EPA
58	9-HEPE	0.386	1.16	Yes	OXOPDAZWPWFJEW-FPRWAWDYSA-N	5283187	R-OH	LOX	EPA
59	5-HEPE	0.706	2.12	Yes	FTAGQROYQYQRHF-FCWZHQICSA-N	6439678	R-OH	LOX	EPA
60	17-HDoHE	0.614	1.84	Yes	SWTYBBUBEPPYCX-VIIQGJSXSA-N	6439179	R-OH	LOX	DHA
61	14-HDoHE	0.545	1.64	Yes	ZNEBXONKCYFJAF-BGKMTWLOSA-N	11566378	R-OH	LOX	DHA
62	4-HDoHE	0.288	0.865	Yes	IFRKCNPQVIJFAQ-JGDWKEERSA-N	53394255	R-OH	LOX	DHA
63	13-KODE	1.27	3.81	Yes	JHXAZBBVQSRKJR-BSZOFBHHSA-N	6446027	R=0	ADH	LA
64	9-KODE	1.13	3.38	Yes	LUZSWWYKKLTDHU-ZJHFMPGASA-N	9839084	R=0	ADH	LA
65	12(13)-Ep-9-KODE	0.812	2.44	No	RCMABBHQYMBYKV-BUHFOSPRSA-N	5283007	R=0	ADH	LA
66	15-KETE	0.723	2.17	No	YGJTUEISKATQSM-USWFWKISSA-N	5280701	R=0	ADH	AA
67	5-KETE	3.38	10.2	No	MEASLHGILYBXFO-XTDASVJISA-N	5283159	R=0	ADH	AA
68	13-HpODE-Screen			No	JDSRHVWSAMTSSN-IRQZEAMPSA-N	5280720	R-OOH	LOX	LA
69	9-HpODE-Screen			No	JGUNZIWGNMQSBM-ZJHFMPGASA-N	6439847	R-OOH	LOX	AA
70	9(10)-EpO	2.59	7.77	Yes	IMYZYCNQZDBZBQ-UHFFFAOYSA-N	15868	Epox	CYP	OA
71	12(13)-EpOME	0.303	0.91	Yes	CCPPLLJZDQAOHD-FLIBITNWSA-N	5356421	Epox	CYP	LA
72	9(10)-EpOME	0.133	0.399	Yes	FBUKMFOXMZRGRB-YFHOEESVSA-N	6246154	Epox	CYP	LA
73	15(16)-EpODE	0.498	1.49	Yes	HKSDVVJONLXYKL-OHPMOLHNSA-N	16061062	Epox	CYP	aLA
74	12(13)-EpODE	0.407	1.22	Yes	BKKGUKSHPCTUGE-OOHFSOINSA-N	16061061	Epox	CYP	aLA
75	9(10)-EpODE	0.443	1.33	Yes	JTEGNNHWOIJBJZ-ZJSQCTGTSA-N	16061060	Epox	CYP	aLA
76	14(15)-EpETrE	0.19	0.57	Yes	WLMZMBKVRPUYIG-LTCHCNGXSA-N	11954058	Epox	CYP	AA
77	11(12)-EpETrE	0.2	0.599	Yes	DXOYQVHGIODESM-IQCOFVSKSA-N	53480479	Epox	CYP	AA

Continuation of **Table S1**.

Report Order	Analyte-ID	LOD (NM)	LOQ (NM)	Observed	InChIKey	PubChem CID	Chemical class	Enzyme	Parent FA
78	8(9)-EpETrE	4.13	12.4	No	DBWQSCSXHFNTMO-TYAUOURKSA-N	5283203	Epox	CYP	AA
79	17(18)-EpETE	0.0844	0.253	No	GPQVVJQEBXAKBJ-JPURVOHMSA-N	16061089	R-OH	LOX	EPA
80	14(15)-EpETE	0.556	1.67	No	RGZIXZYRGZWDMI-QXBXTPPVSA-N	16061088	R-OH	COX	EPA
81	11(12)-EpETE	0.65	1.95	No	QHOKDYBJJBDJGY-BVILWSOJSA-N	16061087	Epox	CYP	EPA
82	19(20)-EpDoPE	0.697	2.09	No	OSXOPUBJJDUAOJ-MBYQGORISA-N	11631565	Epox	CYP	DHA
83	16(17)-EpDoPE	0.514	1.54	No	BCTXZWCPBLWCRV-ZYADFMMDSA-N	14392758	Epox	CYP	DHA
84	15-HETE-EA	0.105	0.314	No	XZQKRCUYLKDPEK-BPVVGZHASA-N	91886095	R-OH	LOX	AA
85	11(12)-EpETre-EA	0.06	0.18	No	TYRRSRADDAROSO-KROJNAHFSA-N	16061183	Epox	CYP	AA
					**PUFA**				
86	LA-Screen			Yes	OYHQOLUKZRVURQ-HZJYTTRNSA-N	5280450	PUFA	Diet	LA
87	ALA-Screen			Yes	DTOSIQBPPRVQHS-PDBXOOCHSA-N	5280934	PUFA	Diet	aLA
88	AA-Screen			Yes	YZXBAPSDXZZRGB-DOFZRALJSA-N	444899	PUFA	D5D	AA
89	EPA-Screen			Yes	JAZBEHYOTPTENJ-JLNKQSITSA-N	446284	PUFA	D6D	EPA
90	DHA-Screen			Yes	MBMBGCFOFBJSGT-KUBAVDMBSA-N	445580	PUFA	D6D	DHA
					**Endocannabinoids**				
91	10-Nitrolinoleate	0.381	1.14	No	LELVHAQTWXTCLY-XYWKCAQWSA-N	5282259	Nitro-FA	NOS	LA
92	9-Nitrooleate	1.18	3.54	No	CQOAKBVRRVHWKV-UHFFFAOYSA-M	53412232	Nitro-FA	NOS	OA
93	10-Nitrooleate	0.565	1.7	No	WRADPCFZZWXOTI-UHFFFAOYSA-N	53394576	Nitro-FA	NOS	OA
94	SEA	43	129	No	OTGQIQQTPXJQRG-UHFFFAOYSA-N	27902	Acyl-EA	PLD	SA
95	PEA	4.75	14.2	No	HXYVTAGFYLMHSO-UHFFFAOYSA-N	4671	Acyl-EA	PLD	PA
96	OEA	0.193	0.58	Yes	BOWVQLFMWHZBEF-KTKRTIGZSA-N	5283454	Acyl-EA	PLD	OA
97	LEA	0.157	0.472	Yes	KQXDGUVSAAQARU-HZJYTTRNSA-N	5283446	Acyl-EA	PLD	LA
98	aLEA	0.0796	0.239	Yes	HBJXRRXWHSHZPU-PDBXOOCHSA-N	5283449	Acyl-EA	PLD	aLA
99	DGLEA	0.149	0.447	Yes	ULQWKETUACYZLI-QNEBEIHSSA-N	5282272	Acyl-EA	PLD	DGLA
100	AEA	0.133	0.399	Yes	LGEQQWMQCRIYKG-DOFZRALJSA-N	5281969	Acyl-EA	PLD	AA
101	DEA	0.398	1.19	Yes	FMVHVRYFQIXOAF-DOFZRALJSA-N	5282273	Acyl-EA	PLD	Adrenic acid
102	DHEA	0.0755	0.227	Yes	CXWASNUDKUTFPQ-KUBAVDMBSA-N	53245830	Acyl-EA	PLD	DHA
103	POEA-Screen			Yes	WFRLANWAASSSFV-FPLPWBNLSA-N	9835868	Acyl-EA	PLD	POA
104	EPEA-Screen			Yes	OVKKNJPJQKTXIT-JLNKQSITSA-N	5283450	Acyl-EA	PLD	EPA
105	NO-Gly	0.156	0.469	Yes	HPFXACZRFJDURI-KTKRTIGZSA-N	6436908	Acyl-Gly	FAAH	OA
106	NA-Gly	0.164	0.491	Yes	YLEARPUNMCCKMP-DOFZRALJSA-N	5283389	Acyl-Gly	FAAH	AA
107	1-LG	2.89	8.66	Yes	WECGLUPZRHILCT-GSNKCQISSA-N	6436630	MAG	Lipase	LA
108	2-LG	1.2	3.61	Yes	IEPGNWMPIFDNSD-HZJYTTRNSA-N	5365676	MAG	Lipase	LG
109	1-AG	0.287	0.862	Yes	DCPCOKIYJYGMDN-HUDVFFLJSA-N	16019980	MAG	Lipase	AA
110	2-AG	0.565	1.69	Yes	RCRCTBLIHCHWDZ-DOFZRALJSA-N	5282280	MAG	Lipase	AA
111	1-OG	2.67	8.01	Yes	RZRNAYUHWVFMIP-QJRAZLAKSA-N	12178130	MAG	Lipase	OA
112	2-OG	1.06	3.19	Yes	UPWGQKDVAURUGE-KTKRTIGZSA-N	5319879	MAG	Lipase	OA
					**NSAID**				
113	Ibuprofen	2.79	8.38	Yes	HEFNNWSXXWATRW-UHFFFAOYSA-N	3672	NSAID	Treatment	NSAID
114	Aspirin			Yes	BSYNRYMUTXBXSQ-UHFFFAOYSA-N	2244	NSAID	Treatment	NSAID
115	Naproxen	9.07	27.2	Yes	CMWTZPSULFXXJA-VIFPVBQESA-N	156391	NSAID	Treatment	NSAID
116	Acetaminophen	2.47	7.4	Yes	RZVAJINKPMORJF-UHFFFAOYSA-N	1983	NSAID	Treatment	NSAID

Continuation of **Table S1**.

Report Order	Analyte ID	LOD (nM	LOQ (nM)	Observed	InChI Key	PubChem_CID	Chemical class	Enzyme	Precursor
**Bile Acids**									
1	CA	1.41	4.23	No	BHQCQFFYRZLCQQ-OELDTZBJSA-N	221493	1º-BA	Cyp27A1; CYP8B1	Cholesterol
2	CDCA	2.03	6.1	Yes	RUDATBOHQWOJDD-BSWAIDMHSA-N	10133	1º-BA	Cyp27A1	Cholesterol
3	UDCA	1.15	3.46	Yes	RUDATBOHQWOJDD-UZVSRGJWSA-N	31401	2º-BA	Microbiome	CDCA
4	DCA	1.36	4.08	Yes	KXGVEGMKQFWNSR-LLQZFEROSA-N	222528	2º-BA	Microbiome	1º-BA Conj
5	LCA	9.43	28	No	SMEROWZSTRWXGI-HVATVPOCSA-N	9903	2º-BA	Microbiome	1º-BA Conj
6	w-MCA	4.97	14.9	Yes	DKPMWHFRUGMUKF-NTPBNISXSA-N	5283851	1º-BA	Cyp27A1	CDCA
7	a-MCA	5.52	16.6	Yes	DKPMWHFRUGMUKF-JDDNAIEOSA-N	53477700	1º-BA	Cyp27A1	CDCA; b-MCA
8	b-MCA	2.64	7.91	Yes	DKPMWHFRUGMUKF-CRKPLTDNSA-N	5283853	1º-BA	Cyp27A1	a-MCA
9	TCA	0.659	1.98	Yes	WBWWGRHZICKQGZ-HZAMXZRMSA-N	6675	1º-BA Conj	BAT	CA
10	TCDCA	0.283	0.846	Yes	BHTRKEVKTKCXOH-BJLOMENOSA-N	387316	1º-BA Conj	BAT	CDCA
11	TDHCA			No	UBDJSBRKNHQFPD-PYGYYAGESA-N	121933	Pre-1º-BA Conj	BAT	DHCA
12	TUDCA	0.047	0.143	Yes	BHTRKEVKTKCXOH-LBSADWJPSA-N	9848818	2º-BA Conj	BAT	UDCA
13	TDCA	0.143	0.43	Yes	AWDRATDZQPNJFN-VAYUFCLWSA-N	2733768	2º-BA Conj	BAT	DCA
14	TLCA	1.55	4.66	No	QBYUNVOYXHFVKC-GBURMNQMSA-N	439763	2º-BA Conj	BAT	LCA
15	GCA	1.38	4.17	Yes	RFDAIACWWDREDC-FRVQLJSFSA-N	10140	1º-BA Conj	BAT	CA
16	GCDCA	0.532	1.6	Yes	GHCZAUBVMUEKKP-GYPHWSFCSA-N	12544	1º-BA Conj	BAT	CDCA
17	GUDCA	0.621	1.86	Yes	GHCZAUBVMUEKKP-XROMFQGDSA-N	12310288	2º-BA Conj	BAT	UDCA
18	GDCA	0.356	1.07	Yes	WVULKSPCQVQLCU-BUXLTGKBSA-N	3035026	2º-BA Conj	BAT	DCA
19	GHDCA	0.297	0.891	No	SPOIYSFQOFYOFZ-BRDORRHWSA-N	114611	2º-BA Conj	BAT	HDCA
20	GLCA	0.522	1.57	Yes	XBSQTYHEGZTYJE-OETIFKLTSA-N	115245	2º-BA Conj	BAT	LCA
21	T-w-MCA	0.505	1.52	No	XSOLDPYUICCHJX-SYCKBGHMSA-N	118703092	1º-BA Conj	BAT	w-MCA
22	T-a-MCA	0.505	1.52	Yes	XSOLDPYUICCHJX-QQXJNSDFSA-N	101657566	1º-BA Conj	BAT	a-MCA
23	T-b-MCA	0.561	1.68	No	XSOLDPYUICCHJX-UZUDEGBHSA-N	21124703	1º-BA Conj	BAT	b-MCA

Continuation of the **Table S1.**

Report Order	Analyte ID	LOD (nM	LOQ (nM)	Observed	InChI Key	PubChem_CID	Chemical class	Enzyme	Precursor
**Steroids**									
24	CRTL	0.875	2.63	Yes	JYGXADMDTFJGBT-VWUMJDOOSA-N	5754	Glucocorticoid	11-beta-HSD1 & 2; Cyp11B1	11-Deoxy-CRTL; CRTN
25	CRTN	0.283	0.848	Yes	MFYSYFVPBJMHGN-ZPOLXVRWSA-N	222786	Glucocorticoid	11-beta-HSD 1 & 2	CRTL
26	CRCTN	1.52	4.56	Yes	OMFXVFTZEKFJBZ-HJTSIMOOSA-N	5753	Mineralo corticoid	Cyp11B1	11-Deoxy-CRTN
27	11-Deoxy-CTRL	0.506	1.52	Yes	WHBHBVVOGNECLV-OBQKJFGGSA-N	440707	Glucocorticoid	CYP8B1	17OH-Prog; 11-Deoxy-CRTN
28	E2	4.66	14	No	VOXZDWNPVJITMN-ZBRFXRBCSA-N	5757	Sex Hormones	Aromatase (CYP19A1)	TEST
30	TEST	0.43	1.29	Yes	MUMGGOZAMZWBJJ-DYKIIFRCSA-N	6013	Sex Hormones	3-beta-HSD	11-Deoxy-CRTL; Andorstenedione
31	17OH-Prog	0.357	1.07	Yes	DBPWSSGDRRHUNT-CEGNMAFCSA-N	6238	Corticosteroid	3-beta-HSD	PROG
32	PROG	0.379	1.14	No	RJKFOVLPORLFTN-LEKSSAKUSA-N	5994	Sex Hormones	3-beta-HSD	Pregnenalone
