# Supplementary material for: Serum metabolomic biomarkers of perceptual speed in cognitively normal and mildly impaired subjects with fasting state stratification
Source: Sci Rep. 2021 Sep 23;11:18964. doi: 10.1038/s41598-021-98640-2 (PMC8460824; doi:10.1038/s41598-021-98640-2)
Supplement: Supplementary file 7 — Supplementary Information 7. [file 41598_2021_98640_MOESM7_ESM.pdf]

Title: Serum metabolomic biomarkers of perceptual speed in cognitively normal and mildly impaired subjects with fasting state stratification

Authors: Kamil Borkowski, Ameer Y. Taha, Theresa L. Pedersen, Philip L. De Jager, David A. Bennett, Rima Kaddurah-Daouk, John W. Newman

Supplemental Table S3. Spearman ρ correlation matrix between serum lipid mediators and cognitive domains. Correlation was generated using subjects stratified by the fasting status. Significant correlations (p<0.04) are highlighted in blue. The numbers indicate Spearman ρ. Percent of metabolites manifesting significant correlation with cognitive domains are indicated above the table.

						Fasted						Non Fasted					
					**% OxyEndo**	4%	2%	2%	8%	2%	2%	1%	1%	0%	10%	2%	5%
					**% Bile Acids**	9%	6%	6%	3%	3%	0%	38%	25%	0%	16%	25%	0%
					**% steroids**	0%	0%	0%	0%	0%	0%	0%	0%	25%	0%	0%	0%
					**Tatal**	5%	3%	3%	6%	2%	2%	10%	7%	2%	10%	8%	3%
**General class**	**Chemical class**	**Enzyme**	**Parent FA**	**Units**	**Variable**	**Episodic memory**	**Global cognition**	**Perceptual orientation**	**Perceptual speed**	**Semantic memory**	**Working memory**	**Spearman ρ Non-Fasted cogn_ep**	**Spearman ρ Non-Fasted cogn_global**	**Spearman ρ Non-Fasted cogn_po**	**Spearman ρ Non-Fasted cogn_ps**	**Spearman ρ Non-Fasted cogn_se**	**Spearman ρ Non-Fasted cogn_wo**
OxyEndo	TX	COX1	AA	nM	TXB2	0.017	-0.054	0.0005	0.08	**-0.25**	-0.079	0.066	0.1	0.074	0.12	**0.2**	-0.031
OxyEndo	PGs	COX2	AA	nM	PGE2	0.098	-0.025	-0.052	0.064	-0.17	-0.11	-0.0052	0.03	-0.06	0.084	0.15	0.026
OxyEndo	PGs	COX2	AA	nM	PGD2	0.14	0.16	0.12	**0.25**	0.088	0.021	0.0059	0.052	-0.024	-0.054	0.17	0.028
OxyEndo	PGs	COX2	AA	nM	PGF2a	0.18	0.18	0.17	0.13	0.04	0.031	-0.018	-0.028	0.024	0.055	-0.051	-0.11
OxyEndo	PGs	COX2	EPA	nM	PGE3	-0.082	-0.046	-0.028	-0.099	-0.015	-0.021	0.078	0.052	-0.0075	-0.053	-0.0094	0.052
OxyEndo	PGs	Auto-ox	AA	nM	F2-IsoP	0.13	0.17	0.19	0.1	0.12	0.12	-0.029	-0.0034	0.039	0.087	-0.074	-0.047
OxyEndo	PG-Gly	COX2	AA	nM	PGF2a-1G	0.03	-0.024	**-0.3**	-0.075	0.048	-0.063	-0.091	-0.087	-0.05	0.031	-0.071	-0.088
OxyEndo	Diol	LOX	AA	nM	LTB4	**-0.31**	-0.23	-0.041	-0.032	-0.2	-0.03	0.043	0.045	-0.042	0.039	-0.023	0.068
OxyEndo	Diol	LOX	AA	nM	5_15-DiHETE	-0.088	-0.032	0.084	-0.0091	-0.028	0.06	-0.0058	0.043	0.14	0.021	0.094	-0.049
OxyEndo	Triol	LOX	LA	nM	9_12_13-TriHOME	-0.065	-0.091	-0.19	-0.059	-0.21	0.012	0.11	0.076	0.059	0.075	0.031	-0.13
OxyEndo	vic-Diol	sEH	OA	nM	9_10-e-DiHO	-0.063	0.017	-0.019	0.061	-0.04	0.13	0.077	0.06	-0.029	0.11	0.074	-0.045
OxyEndo	vic-Diol	sEH	LA	nM	12_13-DiHOME	0.067	0.048	0.044	-0.046	-0.16	0.13	-0.0003	0.0075	0.04	0.033	-0.025	-0.015
OxyEndo	vic-Diol	sEH	LA	nM	9_10-DiHOME	-0.026	-0.067	-0.078	-0.076	-0.22	0.067	0.038	0.016	0.0076	0.015	-0.064	-0.0086
OxyEndo	vic-Diol	sEH	aLA	nM	15_16-DiHODE	0.093	0.062	0.1	-0.089	-0.15	0.15	-0.023	0.0061	0.0012	-0.0086	0.013	-0.028
OxyEndo	vic-Diol	sEH	aLA	nM	12_13-DiHODE	-0.094	-0.088	0.2	-0.15	-0.16	0.028	**0.21**	**0.22**	0.039	0.098	0.098	0.13
OxyEndo	vic-Diol	sEH	aLA	nM	9_10-DiHODE	0.0063	-0.025	-0.09	-0.1	-0.16	0.027	-0.011	0.017	0.019	-0.012	-0.045	0.037
OxyEndo	vic-Diol	sEH	AA	nM	14_15-DiHETrE	0.032	0.023	-0.14	-0.09	-0.04	0.16	-0.013	0.0007	-0.091	0.13	0.13	-0.053
OxyEndo	vic-Diol	sEH	AA	nM	8_9-DiHETrE	-0.085	-0.097	-0.091	-0.13	-0.21	0.069	-0.031	-0.033	-0.059	0.041	0.055	-0.069
OxyEndo	vic-Diol	sEH	AA	nM	5_6-DiHETrE	-0.17	-0.1	0.038	-0.022	-0.075	0.049	0.14	0.16	0.099	0.13	0.11	0.09
OxyEndo	vic-Diol	sEH	EPA	nM	14_15-DiHETE	-0.093	-0.087	0.18	**-0.27**	-0.078	0.15	0.032	0.017	0.012	0.12	-0.08	-0.073
OxyEndo	vic-Diol	sEH	EPA	nM	17_18-DiHETE	-0.053	-0.049	0.11	-0.24	-0.11	0.12	0.04	0.058	-0.035	0.14	0.0042	0.014
OxyEndo	vic-Diol	sEH	DHA	nM	19_20-DiHDoPE	-0.17	-0.18	0.12	**-0.31**	**-0.26**	0.029	0.014	0.063	-0.018	**0.2**	0.079	0.0052
OxyEndo	R-OH	LOX	LA	nM	13-HODE	0.099	0.037	-0.0097	-0.064	-0.23	0.11	-0.0029	0.01	0.038	0.11	-0.02	-0.019
OxyEndo	R-OH	LOX	LA	nM	9-HODE	0.12	0.035	-0.078	-0.035	-0.18	0.073	0.016	0.036	-0.0021	0.12	0.017	0.015
OxyEndo	R-OH	LOX	aLA	nM	13-HOTE	0.08	0.073	0.055	-0.017	-0.1	0.16	-0.028	0.025	0.066	0.088	0.021	-0.0026
OxyEndo	R-OH	LOX	aLA	nM	9-HOTE	0.12	0.081	0.041	-0.085	-0.12	0.13	0.05	0.078	0.0089	0.1	0.066	0.01
OxyEndo	R-OH	CYP	AA	nM	20-HETE	-0.1	0.053	0.0078	0.11	0.18	-0.011	-0.029	-0.073	0.0091	0.0075	0.026	**-0.2**
OxyEndo	R-OH	LOX	AA	nM	15-HETE	0.019	-0.031	-0.026	-0.022	-0.16	-0.026	-0.027	-0.036	0.015	0.017	0.0014	-0.023
OxyEndo	R-OH	LOX	AA	nM	12-HETE	0.0067	-0.049	0.039	-0.09	-0.21	0.0017	0.0035	-0.023	-0.019	-0.043	0.028	-0.0046
OxyEndo	R-OH	LOX	AA	nM	11-HETE	0.15	0.059	-0.073	0.059	-0.14	0.011	-0.022	-0.022	0.042	0.0063	0.077	-0.062
OxyEndo	R-OH	LOX	AA	nM	9-HETE	-0.044	-0.08	-0.21	-0.0013	-0.14	-0.01	-0.089	-0.066	-0.053	0.11	-0.028	-0.044
OxyEndo	R-OH	LOX	AA	nM	5-HETE	-0.13	-0.091	-0.13	0.11	-0.025	0.0046	-0.036	-0.038	0.053	0.046	-0.064	-0.021
OxyEndo	R-OH	LOX	EPA	nM	15-HEPE	0.097	0.16	0.14	0.023	0.073	0.12	0.09	0.12	0.1	**0.2**	0.051	0.011
OxyEndo	R-OH	LOX	EPA	nM	12-HEPE	0.062	0.0017	0.11	-0.15	-0.21	0.038	-0.019	-0.03	-0.011	0.024	-0.011	-0.019
OxyEndo	R-OH	LOX	EPA	nM	5-HEPE	0.068	0.035	0.11	-0.084	-0.0083	-0.017	-0.0053	0.0004	0.11	0.16	-0.053	-0.13
OxyEndo	R-OH	LOX	DHA	nM	17-HDoHE	-0.053	-0.091	0.097	-0.18	-0.21	-0.0028	-0.038	-0.032	0.027	-0.018	0.009	-0.019
OxyEndo	R-OH	LOX	DHA	nM	14-HDoHE	-0.0074	-0.038	0.097	-0.12	-0.21	0.033	-0.018	-0.037	0.025	0.015	-0.014	-0.041
OxyEndo	R-OH	LOX	DHA	nM	4-HDoHE	-0.014	-0.05	0.034	-0.15	-0.069	0.0064	0.073	0.12	0.0038	**0.18**	0.032	0.079
OxyEndo	R=0	ADH	LA	nM	9-KODE	-0.019	0.013	-0.0085	0.067	-0.041	0.11	0.02	0.025	-0.11	0.13	0.05	-0.021
OxyEndo	Epox	CYP	LA	nM	12(13)-EpOME	0.078	0.14	0.099	0.19	-0.012	0.17	0.059	0.11	0.058	0.11	0.031	0.1
OxyEndo	Epox	CYP	LA	nM	9(10)-EpOME	0.018	0.012	0.06	0.017	-0.13	0.13	0.05	0.068	0.074	0.13	-0.016	0.044
OxyEndo	Epox	CYP	aLA	nM	15(16)-EpODE	**0.27**	**0.28**	0.15	0.057	0.017	**0.33**	-0.013	0.023	-0.054	0.074	0.021	0.041
OxyEndo	Epox	CYP	aLA	nM	12(13)-EpODE	-0.13	-0.021	0.04	-0.075	-0.053	0.15	0.073	0.13	-0.033	0.064	**0.18**	0.065
OxyEndo	Epox	CYP	aLA	nM	9(10)-EpODE	-0.06	-0.048	0.015	0.034	-0.095	-0.035	-0.0065	-0.045	-0.07	0.034	-0.083	-0.052
OxyEndo	Epox	CYP	AA	nM	14(15)-EpETrE	0.053	0.08	0.015	0.13	0.062	0.12	0.0082	0.046	0.069	0.097	0.12	-0.012
OxyEndo	PUFA	Diet	LA	Rel Abs	LA_screen	0.005	0.11	0.016	0.1	0.048	0.2	-0.03	0.0014	-0.042	**0.25**	0.1	-0.1
OxyEndo	PUFA	Diet	aLA	Rel Abs	ALA_screen	0.087	0.14	0.07	0.031	0.024	0.12	-0.029	-0.015	-0.02	0.17	0.052	-0.1
OxyEndo	PUFA	D5D	AA	Rel Abs	AA_screen	0.11	0.19	0.13	**0.26**	0.13	0.12	-0.037	-0.09	-0.019	0.1	0.013	**-0.21**
OxyEndo	PUFA	D6D	EPA	Rel Abs	EPA_screen	0.2	0.2	0.22	0.025	0.064	0.099	-0.053	-0.0059	0.037	**0.22**	0.058	-0.14
OxyEndo	PUFA	D6D	DHA	Rel Abs	DHA_screen	0.077	0.12	**0.25**	0.028	0.036	0.036	-0.016	0.026	0.075	**0.25**	0.077	-0.14
OxyEndo	Acyl-EA	PLD	OA	nM	OEA	-0.069	-0.099	-0.035	-0.16	-0.21	0.012	0.034	0.03	0.05	0.1	0.091	-0.11
OxyEndo	Acyl-EA	PLD	LA	nM	LEA	-0.064	-0.11	-0.13	-0.13	-0.1	0.0016	-0.019	-0.023	-0.06	0.046	0.034	-0.03
OxyEndo	Acyl-EA	PLD	aLA	nM	aLEA	-0.1	-0.14	0.039	-0.21	-0.12	-0.063	0.041	0.067	-0.02	0.061	0.12	0.011
OxyEndo	Acyl-EA	PLD	DGLA	nM	DGLEA	-0.15	-0.1	-0.23	-0.073	-0.06	0.033	-0.02	0.031	0.0076	0.16	0.089	0.031
OxyEndo	Acyl-EA	PLD	AA	nM	AEA	-0.098	-0.062	-0.045	-0.064	-0.096	0.086	-0.11	-0.097	-0.015	0.071	-0.064	-0.049
OxyEndo	Acyl-EA	PLD	Adrenic acid	nM	DEA	-0.15	-0.076	-0.095	0.077	-0.073	0.062	0.036	-0.077	-0.035	-0.0032	-0.039	**-0.21**
OxyEndo	Acyl-EA	PLD	DHA	nM	DHEA	-0.027	-0.04	0.11	-0.18	-0.026	-0.027	0.061	0.045	0.018	0.17	-0.032	-0.043
OxyEndo	Acyl-EA	PLD	POA	Rel Abs	POEA_Screen	-0.11	-0.058	-0.016	-0.13	-0.075	0.12	0.018	0.045	0.067	**0.24**	0.032	-0.075
OxyEndo	Acyl-EA	PLD	EPA	Rel Abs	EPEA_Screen	0.053	0.071	-0.0096	-0.12	0.083	0.039	-0.038	-0.018	0.1	**0.18**	-0.036	-0.13
OxyEndo	Acyl-Gly	FAAH	OA	nM	NO-Gly	-0.079	-0.1	-0.0008	-0.12	-0.15	-0.084	0.013	0.012	-0.034	0.13	0.1	-0.052
OxyEndo	Acyl-Gly	FAAH	AA	nM	NA-Gly	0.14	0.16	-0.028	0.23	0.14	0.098	-0.074	-0.087	0.098	0.051	-0.022	-0.13
OxyEndo	MAG	Lipase	LA	nM	1-LG	0.078	0.08	-0.074	0.13	0.053	-0.021	-0.0075	-0.067	-0.055	0.016	-0.03	-0.15
OxyEndo	MAG	Lipase	LG	nM	2-LG	0.069	0.057	-0.077	0.1	0.05	-0.034	-0.034	-0.096	-0.056	-0.012	-0.052	-0.16
OxyEndo	MAG	Lipase	AA	nM	1-AG	0.1	0.082	-0.082	0.16	0.062	-0.067	-0.067	-0.13	-0.043	-0.054	-0.066	-0.15
OxyEndo	MAG	Lipase	AA	nM	2-AG	0.11	0.091	-0.082	0.19	0.078	-0.099	-0.096	-0.15	-0.028	-0.05	-0.074	**-0.18**
OxyEndo	MAG	Lipase	OA	nM	1-OG	0.063	0.055	-0.069	0.12	0.043	-0.049	-0.014	-0.045	-0.01	0.065	0.0006	-0.15
OxyEndo	MAG	Lipase	OA	nM	2-OG	0.09	0.079	0.0049	0.1	0.014	-0.023	0.0024	-0.035	-0.018	0.0097	0.012	-0.1
OxyEndo	-	-	-	nM	Sum_n3_Diols	-0.11	-0.11	0.11	**-0.28**	-0.17	0.1	0.023	0.048	-0.036	0.16	0.018	-0.0041
OxyEndo	-	-	-	nM	Sum(DiHETEs)	-0.052	-0.054	0.12	**-0.25**	-0.12	0.14	0.029	0.049	-0.031	0.15	0.0064	-0.0061
OxyEndo	-	-	-	nM	Sum(HDoHEs)	-0.018	-0.051	0.087	-0.13	-0.22	0.028	-0.012	-0.034	0.024	0.023	-0.012	-0.047
OxyEndo	-	-	-	Ratio	15_16-DiHODE/15(16)-EpODE	**-0.27**	**-0.32**	-0.066	-0.17	-0.2	**-0.28**	0.034	0.05	0.11	-0.045	0.07	-0.044
OxyEndo	-	-	-	Ratio	12_13-DiHOME/12(13)-EpOME	-0.051	-0.16	-0.076	**-0.32**	-0.14	-0.16	-0.11	-0.11	-0.025	-0.018	0.019	-0.15
OxyEndo	-	-	-	Ratio	14_15-DiHETrE/14(15)-EpETrE	-0.055	-0.099	-0.075	-0.24	-0.12	-0.071	-0.0092	-0.052	-0.084	-0.027	-0.084	-0.043
OxyEndo	-	-	-	Ratio	11_12-DiHETrE	-0.024	0.0095	-0.093	-0.075	-0.086	0.22	-0.033	0.012	0.023	0.13	0.14	-0.053
OxyEndo	-	-	-	Ratio	12-HEPE/12-HETE	0.13	0.13	0.17	-0.14	0.028	0.072	-0.064	-0.0078	0.009	0.15	-0.077	0.039
OxyEndo	-	-	-	Ratio	9,10-DiHOME/EpOME 2	0.054	0.059	-0.13	0.018	0.11	0.006	-0.029	-0.095	-0.088	-0.12	-0.044	-0.15
OxyEndo	-	-	-	Ratio	9_10-DiHOME/9(10)-EpOME	0.054	0.059	-0.13	0.018	0.11	0.006	-0.029	-0.095	-0.088	-0.12	-0.044	-0.15
OxyEndo	-	-	-	Ratio	Sum(DiHODE/EpODE)	-0.16	-0.24	-0.019	-0.14	-0.17	-0.23	0.0027	0.036	0.11	-0.043	0.064	-0.032
OxyEndo	-	-	-	Ratio	9-KODE/9-HODE	-0.069	0.039	0.09	0.078	0.15	0.11	0.012	0.015	-0.095	0.033	0.061	-0.021
OxyEndo	-	-	-	Ratio	Sum(DiHETrE/EpETrE)	-0.093	-0.13	-0.076	-0.22	-0.14	-0.092	0.02	-0.015	-0.056	-0.031	-0.056	-0.0044
OxyEndo	-	-	-	Ratio	12_13-DiHODE/12(13)-EpODE	0.057	-0.0019	0.14	0.045	-0.074	-0.054	0.17	0.13	0.11	0.03	-0.067	0.12
OxyEndo	-	-	-	Ratio	14,15/11,12-DiHETrE	0.051	-0.056	-0.1	-0.11	0.031	-0.24	0.078	0.036	-0.12	0.037	-0.002	0.0049

Continuation of **Supplemental Table S2.**

General class	Chemical class	Enzyme	Parent FA	Units	Variable	Episodic memory	Global cognition	Perceptual orientation	Perceptual speed	Semantic memory	Working memory	Spearman ρ Non-Fasted cogn_ep	Spearman ρ Non-Fasted cogn_global	Spearman ρ Non-Fasted cogn_po	Spearman ρ Non-Fasted cogn_ps	Spearman ρ Non-Fasted cogn_se	Spearman ρ Non-Fasted cogn_wo
OxyEndo	-	-	-	Ratio	9_10-DiHODE/9(10)-EpODE	0.1	0.024	-0.056	-0.084	-0.041	-0.04	-0.066	0.015	0.069	0.0007	0.04	0.047
NSAD	NSAID	Treatment		nM	Ibuprofen	-0.13	-0.14	-0.15	0.0081	-0.056	-0.19	-0.1	-0.11	-0.012	-0.0086	-0.05	-0.13
NSAD	NSAID	Treatment		nM	Aspirin	0.044	-0.0008	0.081	0.0069	-0.026	-0.12	0.018	0.051	0.023	0.085	-0.055	0.13
NSAD	NSAID	Treatment		nM	Acetaminophen	-0.052	-0.13	-0.024	-0.046	-0.16	-0.1	0.0038	-0.083	-0.021	-0.14	-0.14	-0.042
Bile Acid	Cyp27A1; CYP8B1	1º-BA	-	nM	CA	0.21	0.17	0.16	0.089	0.057	-0.022	0.15	0.096	-0.11	0.14	0.14	-0.093
Bile Acid	Cholesterol	Cyp27A1	-	nM	CDCA	**0.27**	0.2	0.061	0.11	0.11	-0.047	0.094	0.15	-0.0068	**0.2**	**0.19**	0.043
Bile Acid	CDCA	Microbiome	-	nM	UDCA	-0.023	-0.096	**-0.25**	-0.067	-0.013	-0.13	-0.083	-0.11	0.02	-0.12	-0.047	-0.078
Bile Acid	1º-BA Conj	Microbiome	-	nM	DCA	0.2	0.17	-0.086	0.19	0.21	-0.029	0.014	0.12	0.11	0.078	**0.2**	0.1
Bile Acid	CDCA	Cyp27A1	-	nM	w-MCA	0.091	0.068	-0.029	0.07	0.15	-0.028	0.1	0.14	0.081	0.066	0.15	0.063
Bile Acid	CA	BAT	-	nM	TCA	0.071	0.048	0.08	-0.04	-0.05	0.15	-0.086	-0.096	-0.04	-0.026	-0.11	-0.046
Bile Acid	CDCA	BAT	-	nM	TCDCA	**0.058**	0.006	0.052	-0.1	-0.071	0.11	-0.075	-0.14	-0.1	-0.084	**-0.2**	-0.1
Bile Acid	DCA	BAT	-	nM	TDCA	-0.035	-0.044	0.014	-0.013	-0.03	0.031	**-0.18**	-0.14	0.0071	-0.09	-0.12	-0.016
Bile Acid	BAT	2º-BA Conj	-	nM	TLCA	0.013	-0.033	-0.023	-0.072	-0.11	0.067	**-0.28**	**-0.29**	-0.086	**-0.2**	**-0.27**	-0.059
Bile Acid	CA	BAT	-	nM	GCA	0.18	0.15	0.13	0.018	0.1	0.11	-0.11	-0.073	-0.052	0.027	-0.051	-0.019
Bile Acid	CDCA	BAT	-	nM	GCDCA	0.22	0.19	0.057	0.021	0.18	0.098	-0.05	-0.082	-0.073	-0.025	-0.13	-0.04
Bile Acid	UDCA	BAT	-	nM	GUDCA	0.21	0.17	0.0077	0.015	0.22	0.053	-0.02	-0.045	-0.15	-0.056	-0.014	0.0001
Bile Acid	DCA	BAT	-	nM	GDCA	0.15	0.14	-0.011	0.15	0.16	0.045	**-0.21**	-0.13	0.0098	-0.08	-0.051	0.021
Bile Acid	LCA	BAT	-	nM	GLCA	0.11	0.063	-0.017	-0.012	0.059	-0.024	-0.13	-0.056	-0.037	-0.062	0.016	0.071
Bile Acid	BAT	1º-BA Conj	-	nM	T-a-MCA	**-0.29**	**-0.31**	-0.043	**-0.28**	**-0.26**	-0.066	-0.081	-0.14	-0.067	-0.16	-0.15	0.0093
Bile Acid	-	-	-	Ratio	GUDCA/UDCA	**0.3**	**0.32**	**0.33**	0.081	0.24	0.2	0.047	0.049	-0.12	0.072	0.0068	0.052
Bile Acid	-	-	-	Ratio	GDCA/CA	-0.11	-0.077	-0.14	-0.0008	0.045	0.05	**-0.28**	**-0.18**	0.089	-0.16	-0.13	0.072
Bile Acid	-	-	-	Ratio	TDCA/CA	-0.17	-0.15	-0.13	-0.084	-0.049	0.05	**-0.26**	**-0.19**	0.048	-0.15	-0.17	0.027
Bile Acid	-	-	-	Ratio	TLCA/CDCA	-0.16	-0.14	-0.085	-0.13	-0.14	0.088	**-0.24**	**-0.27**	-0.025	**-0.24**	**-0.28**	-0.057
Bile Acid	-	-	-	Ratio	GDCA/DCA	-0.038	-0.047	0.12	-0.097	-0.093	0.042	**-0.23**	**-0.27**	-0.14	-0.16	**-0.3**	-0.062
Bile Acid	-	-	-	Ratio	GCA/CA	-0.062	-0.057	-0.053	-0.091	0.013	0.093	**-0.22**	-0.15	0.04	-0.092	-0.13	0.075
Bile Acid	-	-	-	Ratio	TCA/CA	-0.1	-0.11	-0.041	-0.11	-0.084	0.071	**-0.21**	-0.16	0.021	-0.099	-0.16	0.018
Bile Acid	-	-	-	Ratio	DCA/CA	-0.034	0.011	-0.2	0.074	0.16	0.091	**-0.19**	-0.062	0.15	-0.089	-0.0091	0.13
Bile Acid	-	-	-	Ratio	TDCA/DCA	-0.14	-0.17	0.049	-0.19	-0.23	0.053	**-0.18**	**-0.22**	-0.08	-0.12	**-0.25**	-0.081
Bile Acid	-	-	-	Ratio	(GDCA+GLCA)/(TDCA+TLCA)	0.2	0.16	0.022	-0.063	0.15	0.017	**0.18**	**0.19**	0.011	0.14	0.15	0.098
Bile Acid	-	-	-	Ratio	GCDCA/CDCA	-0.14	-0.11	-0.0041	-0.22	-0.093	0.18	-0.13	**-0.2**	-0.034	**-0.24**	**-0.28**	-0.054
Bile Acid	-	-	-	Ratio	GLCA/CDCA	-0.17	-0.16	-0.11	-0.16	-0.065	0.032	-0.16	-0.14	0.022	**-0.19**	-0.14	0.027
Bile Acid	-	-	-	Ratio	TDCA/TLCA	-0.029	-0.053	0.0025	0.055	0.012	-0.1	0.064	0.14	0.075	0.1	0.13	0.095
Bile Acid	-	-	-	Ratio	GCA/GDCA	0.077	0.058	0.2	-0.09	-0.06	0.1	0.06	0.006	-0.11	0.094	-0.028	-0.061
Bile Acid	-	-	-	Ratio	CA/CDCA	-0.065	-0.053	0.16	-0.034	-0.15	-0.023	0.11	-0.016	-0.12	-0.0093	0.0069	-0.15
Bile Acid	-	-	-	Ratio	GCDCA/GLCA	0.03	0.033	0.083	-0.0095	-0.023	0.091	0.066	-0.012	-0.0069	0.028	-0.09	-0.09
Bile Acid	-	-	-	Ratio	GDCA/GLCA	0.061	0.064	-0.021	0.13	0.02	0.11	-0.068	-0.029	0.038	0.012	-0.02	0.033
Steroids	11-Deoxy-CRTL; CRTN	11-beta-HSD; Cyp11B1	-	nM	CRTL	-0.021	-0.0032	-0.046	-0.052	0.15	0.021	-0.0089	-0.07	-0.071	-0.066	-0.023	-0.067
Steroids	CRTL	11-beta-HSD	-	nM	CRTN	-0.023	-0.012	-0.016	-0.12	0.024	0.11	-0.015	-0.062	-0.082	-0.071	-0.038	-0.025
Steroids	11-Deoxy-CRTN	Cyp11B1	-	nM	CRCTN	-0.044	-0.06	-0.054	-0.13	0.14	-0.078	-0.0018	-0.051	-0.055	-0.076	0.012	-0.048
Steroids	17OH-Prog; 11-Deoxy-CRTN	CYP8B1	-	nM	11-Deoxy-CTRL	-0.073	-0.016	0.099	0.087	0.059	0.088	0.019	-0.052	-0.065	-0.13	-0.051	-0.11
Steroids	-	-	-	nM	DHEAS	-0.061	-0.01	0.0026	0.0047	0.17	0.067	0.02	0.032	0.0046	0.093	0.019	-0.055
Steroids	11-Deoxy-CRTL; Andorstenedione	3-beta-HSD	-	nM	TEST	0.024	0.074	-0.011	0.039	-0.023	0.23	0.053	0.025	-0.012	-0.014	-0.0075	-0.024
Steroids	PROG	3-beta-HSD	-	nM	17OH-PROG	0.018	0.035	0.092	0.013	0.05	0.11	0.054	0.017	**-0.2**	0.016	0.027	-0.0049
Steroids	-	-	-	Ratio	Tes/Prog	-0.041	0.0086	-0.11	0.028	-0.084	0.18	0.036	0.021	**0.18**	-0.028	-0.042	-0.053
