# Supplementary material for: Serum metabolomic biomarkers of perceptual speed in cognitively normal and mildly impaired subjects with fasting state stratification
Source: Sci Rep. 2021 Sep 23;11:18964. doi: 10.1038/s41598-021-98640-2 (PMC8460824; doi:10.1038/s41598-021-98640-2)
Supplement: Supplementary file 9 — Supplementary Information 9. [file 41598_2021_98640_MOESM9_ESM.pdf]

Title: Serum metabolomic biomarkers of perceptual speed in cognitively normal and mildly impaired subjects with fasting state stratification

Authors: Kamil Borkowski, Ameer Y. Taha, Theresa L. Pedersen, Philip L. De Jager, David A. Bennett, Rima Kaddurah-Daouk, John W. Newman

Supplemental Table S5. Stepwise linear model predictive cognitive domains, stratified by the fasting state. Stepwise analysis was performed with the maximal validation r² as the model stopping criteria, or if an additional step increased the BIC. Model stopping point for each domain is highlighted.

Fasted

Cognitive domain	Step	Parameter	Sig Prob	Seq SS	RSquare	AICc	BIC	RSquare Validation
**Episodic memory**	1	**Johnson Su[LTB4] 2**	**0.0083**	2.34	0.174	68.4	72.7	-0.12
	2	**Johnson Su[T-a-MCA]**	**0.0214**	1.54	0.288	65.1	70.6	-0.0568
	3	Johnson Sb[GUDCA/UDCA]	0.131	0.613	0.334	65.2	71.7	0.127
	4	Johnson Su[19_20-DiHDoPE] 2	0.299	0.285	0.355	66.7	74.1	0.14
**Perceptual orientation**	1	**Johnson Sb[TDCA/CA]**	**0.0106**	2.34	0.164	71.2	75.5	-0.0762
	2	**Johnson Sb[GUDCA/UDCA]**	**0.0729**	1.03	0.236	70.1	75.6	0.0806
	3	**Johnson Su[PGF2a-1G] 2**	**0.036**	1.3	0.328	67.8	74.3	0.222
	4	**PGE2/PGD2**	**0.0572**	0.982	0.397	66.4	73.8	0.229
	5	Johnson Su[9-HETE] 2	0.156	0.517	0.433	67	75	0.254
	6	Johnson Su[GDCA/DCA]	0.186	0.437	0.463	68	76.5	0.249
**Perceptual speed**	1	**Johnson Sb[12,1...DiHOME/EpOME 2]**	**0.0014**	5	0.219	91.7	96.5	-0.027
	2	**Johnson Su[Sum_n3_Diols]**	**0.0018**	3.81	0.386	83.6	89.7	0.0919
	3	**Johnson Su[T-a-MCA]**	**0.0462**	1.34	0.445	81.7	89	0.235
	4	Johnson Su[PGF2a]	0.38	0.251	0.456	83.5	91.9	0.302
	5	Johnson Su[19_20-DiHDoPE]	0.585	0.0984	0.46	86	95.4	0.34
**Semantic memory**	1	Johnson Su[17-HDoHE]	0.0458	1.36	0.104	70.8	75.1	-0.315
	2	Johnson Su[T-a-MCA]	0.0232	1.6	0.225	67.6	73.1	-0.332
	3	Johnson Sb[TXB2]	0.0638	0.968	0.298	66.4	72.9	-0.477
**Working memory**	1	**Johnson Su[CRTN]**	0.127	0.922	0.062	77.3	81.6	0.0093
	2	Johnson Su[15_1...E/15(16)-EpODE]	0.206	0.615	0.103	78	83.5	0.0736
	3	Johnson Sb[ALA_screen]	0.407	0.264	0.121	79.9	86.4	-0.0302
**Global cognition**	1	Johnson Sb[GUDCA/UDCA]	**0.0244**	1.23	0.115	64	68.7	-0.252
	2	**Johnson Su[T-a-MCA]**	**0.0137**	1.32	0.238	59.8	65.9	0.127
	3	Johnson Su[15(16)-EpODE]	0.101	0.539	0.289	59.4	66.7	-0.0067
	4	Johnson Su[19_20-DiHDoPE]	0.165	0.373	0.323	59.8	68.3	0.0578

Non-fasted

Cognitive domain	Step	Parameter	Sig Prob	Seq SS	RSquare	AICc	BIC	RSquare Validation
**Episodic memory**	1	**Johnson Sb[TLCA]**	**0.0003**	3.94	0.144	139	146	-0.011
	2	Johnson Sb[4-HDoHE]	**0.0258**	1.35	0.194	136	145	-0.196
	3	Johnson Su[12_1...E/12(13)-EpODE]	0.153	0.538	0.213	136	148	-0.142
**Perceptual orientation**	1	**Johnson Sb[DCA/CA]**	**0.0573**	1.7	0.0419	183	190	-0.0227
	2	Johnson Sb[TDCA/TLCA]	0.0581	1.64	0.0822	182	191	-0.1
	3	Johnson Sb[5-HEPE]	0.14	0.973	0.106	182	193	-0.12
**Perceptual speed**	1	**Johnson Su[LA_screen]**	**0.0071**	3.98	0.0821	195	202	-0.128
	2	Johnson Sb[4-HDoHE]	**0.0343**	2.32	0.13	192	202	-0.218
	3	Johnson Sb[TXB2]	0.232	0.724	0.145	193	205	-0.291
**Semantic memory**	1	**Johnson Su[GDCA/DCA]**	**0.0002**	3.59	0.153	125	132	-0.107
	2	Johnson Su[12(13)-EpODE]	**0.0402**	0.978	0.194	123	132	-0.0327
	3	Johnson Sb[TLCA]	0.104	0.597	0.22	122	134	0.002
**Working memory**	1	**Johnson Sb[AA_screen]**	**0.0243**	2.08	0.0583	170	177	0.0233
	2	Johnson Su[(GDC...A)/(TDCA+TLCA)]	0.0594	1.4	0.0975	169	178	-0.0345
	3	Johnson Sb[Tes/Prog]	0.198	0.641	0.116	169	181	-0.114
**Global cognition**	1	**Johnson Sb[TLCA]**	**0.0002**	2.36	0.152	89.2	96.4	-0.104
	2	Johnson Su[12_13-DiHODE]	**0.0475**	0.607	0.191	87.4	96.7	-0.0725
	3	Johnson Su[GDCA/DCA]	0.274	0.181	0.202	88.3	99.9	-0.0481
