# Supplementary material for: Serum metabolomic biomarkers of perceptual speed in cognitively normal and mildly impaired subjects with fasting state stratification
Source: Sci Rep. 2021 Sep 23;11:18964. doi: 10.1038/s41598-021-98640-2 (PMC8460824; doi:10.1038/s41598-021-98640-2)
Supplement: Supplementary file 10 — Supplementary Information 10. [file 41598_2021_98640_MOESM10_ESM.pdf]

Title: Serum metabolomic biomarkers of perceptual speed in cognitively normal and mildly impaired subjects with fasting state stratification

Authors: Kamil Borkowski, Ameer Y. Taha, Theresa L. Pedersen, Philip L. De Jager, David A. Bennett, Rima Kaddurah- Daouk, John W. Newman

Supplemental Table S6. Variables preselected for PS stepwise model, clustered using JMP PCA algorithm.

Cluster	Members	RSquare with Own Cluster	RSquare with Next Closest	1-RSquare Ratio
1	Sum_n3_Diols	0.97	0.023	0.032
1	17,18-DiHETE +19,20-DiHDoPe	0.96	0.024	0.041
1	Sum(DiHETEs)	0.9	0.025	0.099
1	19_20-DiHDoPE	0.7	0.017	0.31
1	aLEA	0.23	0.039	0.8
1	AA	0.072	0.017	0.94
2	GCDCA/CDCA	0.7	0.017	0.3
2	T-a-MCA	0.67	0.013	0.34
2	NA-Gly	0.42	0.033	0.6
3	12,13-DiHOME/EpOME	0.51	0.027	0.51
3	PGE3	0.51	0.039	0.51
